# A thalamic circuit facilitates stress susceptibility via melanocortin 4 receptor‐mediated activation of nucleus accumbens shell

**DOI:** 10.1111/cns.14046

**Published:** 2022-12-12

**Authors:** Qiao Deng, Shao‐Qi Zhang, Ping‐Fen Yang, Wan‐Ting Dong, Jia‐Lin Wang, Jian‐Guo Chen, Fang Wang, Li‐Hong Long

**Affiliations:** ^1^ Department of Pharmacology School of Basic Medicine, Tongji Medical College, Huazhong University of Science and Technology Wuhan City Hubei China; ^2^ The Research Center for Depression Tongji Medical College, Huazhong University of Science and Technology Wuhan China; ^3^ The Key Laboratory for Drug Target Researches and Pharmacodynamic Evaluation of Hubei Province Wuhan China; ^4^ Key Laboratory of Neurological Diseases (HUST) Ministry of Education of China Wuhan City Hubei China; ^5^ Laboratory of Neuropsychiatric Diseases The Institute of Brain Research, Huazhong University of Science and Technology Wuhan China

**Keywords:** excitatory neurotransmission, melanocortin 4 receptor, nucleus accumbens shell, paraventricular thalamus, stress susceptibility

## Abstract

**Aims:**

Central melanocortin 4 receptor (MC4R) has been reported to induce anhedonia via eliciting dysfunction of excitatory synapses. It is evident that metabolic signals are closely related to chronic stress‐induced depression. Here, we investigated that a neural circuit is involved in melanocortin signaling contributing to susceptibility to stress.

**Methods:**

Chronic social defeat stress (CSDS) was used to develop depressive‐like behavior. Electrophysiologic and chemogenetic approaches were performed to evaluate the role of paraventricular thalamus (PVT) glutamatergic to nucleus accumbens shell (NAcsh) circuit in stress susceptibility. Pharmacological and genetic manipulations were applied to investigate the molecular mechanisms of melanocortin signaling in the circuit.

**Results:**

CSDS increases the excitatory neurotransmission in NAcsh through MC4R signaling. The enhanced excitatory synaptic input in NAcsh is projected from PVT glutamatergic neurons. Moreover, chemogenetic manipulation of PVT^Glu^‐NAcsh projection mediates the susceptibility to stress, which is dependent on MC4R signaling. Overall, these results reveal that the strengthened excitatory neurotransmission in NAcsh originates from PVT glutamatergic neurons, facilitating the susceptibility to stress through melanocortin signaling.

**Conclusions:**

Our results make a strong case for harnessing a thalamic circuit to reorganize excitatory synaptic transmission in relieving stress susceptibility and provide insights gained on metabolic underpinnings of protection against stress‐induced depressive‐like behavior.

## Graphical abstract

The schematic representation of the proposed model describing the PVT‐NAcsh circuit diagram encountered with social stress. CSDS induces activation of MC4R signaling and consequently strengthens excitatory synaptic transmission in NAcsh. Furthermore, the enhanced excitatory synaptic input in NAcsh originates from PVT glutamatergic neurons, which is dependent on MC4R signaling. This process participates in susceptibility to stress.

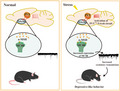

## 1 INTRODUCTION

A variety of metabolic signals in charge of feeding have been implicated in mood regulation by exerting an effect on the central nervous system.^1^, ^2^ Central melanocortin 4 receptor (MC4R) has been recognized as a suppressor of appetite and plays an essential role in anhedonia, anxiety, and depression,^3^, ^4^ which is activated by its endogenous agonist, α‐melanocyte‐stimulating hormone (α‐MSH).^5^, ^6^ Furthermore, both exogenous MC4R antagonist and genetic knockdown of MC4R in central nervous system ameliorate depressive‐like behavior induced by chronic stress.^3^, ^4^, ^7^

Our previous studies have reported the dysfunction of postsynaptic glutamatergic transmission in multiple brain areas from chronic social defeat stress (CSDS)‐treated mice.^8^, ^9^, ^10^, ^11^ There are several clues that MC4R signaling in the nucleus accumbens (NAc) mediates excitatory postsynaptic adaptation and triggers anhedonia.^4^ For example, due to the activation of MC4R in NAc, the strength of excitatory synapses is decreased in dopamine D_1_ receptor‐expressing medium spiny neurons after exposure to chronic stress. Although postsynaptic melanocortin signaling has been implicated in depression, the neural circuit underlying stress‐induced depressive‐like behavior that is involved in melanocortin signaling remains poorly understood.

Neurons in NAc, including NAc shell (NAcsh) and NAc core (NAcc),^12^, ^13^ receive the primary glutamatergic input from the paraventricular thalamus (PVT).^14^ It is worth noting that a variety of concomitant symptoms in depressed patients, such as somnipathy, aversion, and maladaptive feeding, including both hyper‐ and hypophagia.^15^, ^16^ Furthermore, PVT has been originally well recognized as regulating hub in feeding behavior.^17^, ^18^, ^19^ Emerging evidence reveals that the activation of PVT neurons has also been related to stress‐induced behavior.^20^ For example, both chronic stress, such as foot shock, and acute stress, including forced swim and restraint stress, result in the elevated activity of PVT neurons.^21^, ^22^ Recent studies have found that PVT‐related circuits are involved in chronic pain‐induced anxiety and other forms of anxiety.^23^, ^24^ In addition, optogenetic activation of PVT‐NAc circuit promotes wakefulness, morphine withdrawal symptoms,^14^, ^25^ or inhibition of PVT‐NAc circuit enhances drug addiction, such as cocaine preference and heroin relapse.^26^, ^27^ Neuroimaging study has shown the abnormal neural connectivity of PVT in depressed patients.^22^ Moreover, the activity of PVT neurons could be reduced by classic antidepressant fluoxetine.^28^ Although PVT‐projecting neurons have been implicated in mediating other behavior, little is known about whether the synaptic input from PVT projection to NAc has an essential role in stress‐induced depressive‐like behavior. In the present study, we found that activation of MC4R in NAcsh strengthened the excitatory synaptic transmission induced by CSDS. Furthermore, we identified the excitatory projections from PVT onto NAcsh as the neural circuit promoting MC4R‐mediated susceptibility to stress. Our findings provide new insights into the amelioration of stress susceptibility via the thalamic circuit and help form a metabolic basis for protection against stress‐induced depressive‐like behavior.

## 2 MATERIALS AND METHODS

### 2.1 Animals

All experiments conformed to the National Institutes of Health Guide for the Care and Use of Laboratory Animals. All animal procedures were approved by the Animal Welfare Committee of Huazhong University of Science and Technology and followed by ARRIVE 2.0 guidelines.^29^ C57BL/6J (Hunan SJA Laboratory Animal Corporation Ltd, Changsha, China) male mice (8–13 weeks old) of normal appearance and body weight were used for all behavioral assessments, immunofluorescent, and electrophysiology experiments. CD1 retired breeders (Beijing Vital River Laboratory Animal Technology Co., Ltd. Beijing, China) were singly housed. All animals were housed in a 12‐h light/dark at a constant temperature (22 ± 2°C) with water and food ad libitum.

### 2.2 Chronic social defeat stress (CSDS)

Chronic social defeat stress was carried out as previously reported.^30^ CD1 resident mice were screened by the latency of aggression. Each experimental male C57BL/6J mouse was introduced into the home cage of a novel aggressive CD1 resident mouse for 5–10 min and physically defeated daily over 10 consecutive days. Then, the intruder and resident mouse were housed in one‐half of the home cage separated by perforated plexiglass to maintain sensory stress for 24 h. Control mice were housed in pairs under the same conditions but without the presence of an aggressive CD1 resident mouse. Twenty‐four hours after the final social defeat, the defeated mice and control mice were housed singly.

### 2.3 Subthreshold social defeat stress (SSDS)

Subthreshold social defeat stress was carried out as previously reported.^31^ Experimental mice were placed into the home cage of a resident aggressive CD1 for 5 min and subjected to physical attack. Then, the intruder and the aggressor were separated by the perforated plexiglass for 15 min, this operation was performed as above three times. After the subthreshold defeat, experimental mice were detected 24 h later for behavioral assessments.

### 2.4 Behavioral assessments

After social defeat stress, experimental mice were subjected to behavioral assessments, including social interaction test (SIT), sucrose preference test (SPT), tail suspension test (TST), forced swim test (FST), and locomotor activity.

### 2.5 Drugs

DREADD (designer receptors exclusively activated by designer drug) agonist Clozapine‐N oxide (CNO) (5 μM)^32^ was dissolved in 0.1% DMSO and purchased from MedChen Express (NJ, USA) and stereotaxic microinjected a volume of 1 μl into NAcsh through the stainless‐steel guide cannulas with a 5 μl syringe. MC4R antagonist SHU9119 (50 μM, Abcam, Cambridge, UK)^19^ was dissolved in PBS.

### 2.6 Electrophysiology

Whole‐cell patch‐clamp recordings. Mice were deeply anesthetized with isoflurane and perfumed with 40 ml ice oxygenated (95% O_2_ and 5% CO_2_) solution containing (in mM), 210 sucrose, 3.1 sodium pyruvate, 11.6 L‐ascorbate, 1.0 NaH_2_PO_4_, 26.2 NaHCO_3_, MgCl_2_, and 20.0 glucose (pH 7.4, 300 mOsm). And then, brains were cut into coronal PVT or NAcsh‐containing slices (300 μm) using a vibratome (VT1000S, Leica, Wetzlar, Germany). Incubation of slices for at least 1 h at room temperature was performed in the oxygenated (95% O_2_ and 5% CO_2_) artificial cerebrospinal fluid (ACSF) containing (in mM), 119.0 NaCl, 3.5 KCl, 1.3 MgSO_4_, 1.0 NaH_2_PO_4_, 26.2 NaHCO_3_, 11.0 glucose, and 2.5 CaCl_2_ (pH 7.4, 300 mOsm).

Voltage‐clamp mode. Brain sections were transferred into the recording chamber with circulating ACSF at room temperature. Using an electrode (3–6 MΩ resistance) filled with intracellular solution containing (in mM), 122.5 Cs‐gluconate, 17.5 CsCl, 0.2 EGTA, 10.0 HEPES, 1.0 MgCl_2_ 0.3 Na‐GTP, 5 QX314 (pH 7.2, 280–300 mOsm), we recorded α‐amino‐3‐hydroxy‐5‐methyl‐4‐isoxazolpropionic acid receptor (AMPAR)‐mediated miniature excitatory postsynaptic currents (mEPSCs) in NAcsh neurons at a holding potential of −70 mV by the MultiClamp 700B amplifier (Molecular Devices, Sunnyvale, CA). Meanwhile, we added tetrodotoxin (TTX) (1 μM, Sigma‐Aldrich, MO, USA) and bicuculline (20 μM, Sigma‐Aldrich, MO, USA) into an incubated slice chamber to isolate mEPSCs. To examine the effect of α‐MSH‐MC4R on excitatory synaptic transmission induced by CSDS, the NAcsh‐containing brain slices were incubated with α‐MSH (1 μM, Abcam, ab120189, Cambridge, UK)^4^ or SHU9119 (50 μM) for 2 h, respectively.

Current‐clamp mode. We recorded action potential (AP) of PVT neurons with the injection current of 0–140 pA at a holding potential of −70 mV. We used the internal solution that contained (in mM), 97 K‐glucomate, 38 KCl, 0.35 EGTA, 20 HEPES, 6 NaCl, 7 Phosphocreatine‐Na, 4 Mg‐ATP, 0.35 Na‐GTP (pH 7.2280–300 mOsm). All recordings were performed under an upright Olympus microscope (BX51WIF, Olympus, Tokyo, Japan). A Digidata 1322A digitizer (Molecular Devices) was used to filter data at 2 kHz and 10 kHz. Data analysis was performed by a mini analysis program (Synaptosoft, GA, USA).

Detailed information for behavioral assessments, western blotting, immunofluorescence, viral injection, and cannula implantation are provided in Appendix S1.

### 2.7 Statistical analysis

Animals were randomly allocated to groups, and the analysis was conducted by a blind individual. Data were expressed as mean ± SEM and analyzed by using GraphPad 6.0 software (GraphPad, CA, USA). Data were analyzed for normal distribution with the Shapiro–Wilk test. Comparison between the two groups was employed by unpaired Student's *t* test. Significance of multiple variables was used one‐ or two‐way ANOVA followed by Bonferroni's post hoc. If data do not exhibit a normal distribution, statistical significance was analyzed by the Mann–Whitney test and Kruskal–Wallis one‐way analysis of variance. Statistical significance was defined as *p* < 0.05.

## 3 RESULTS

### 3.1 CSDS increases excitatory synaptic transmission in NAcsh through MC4R

**FIGURE 1:**
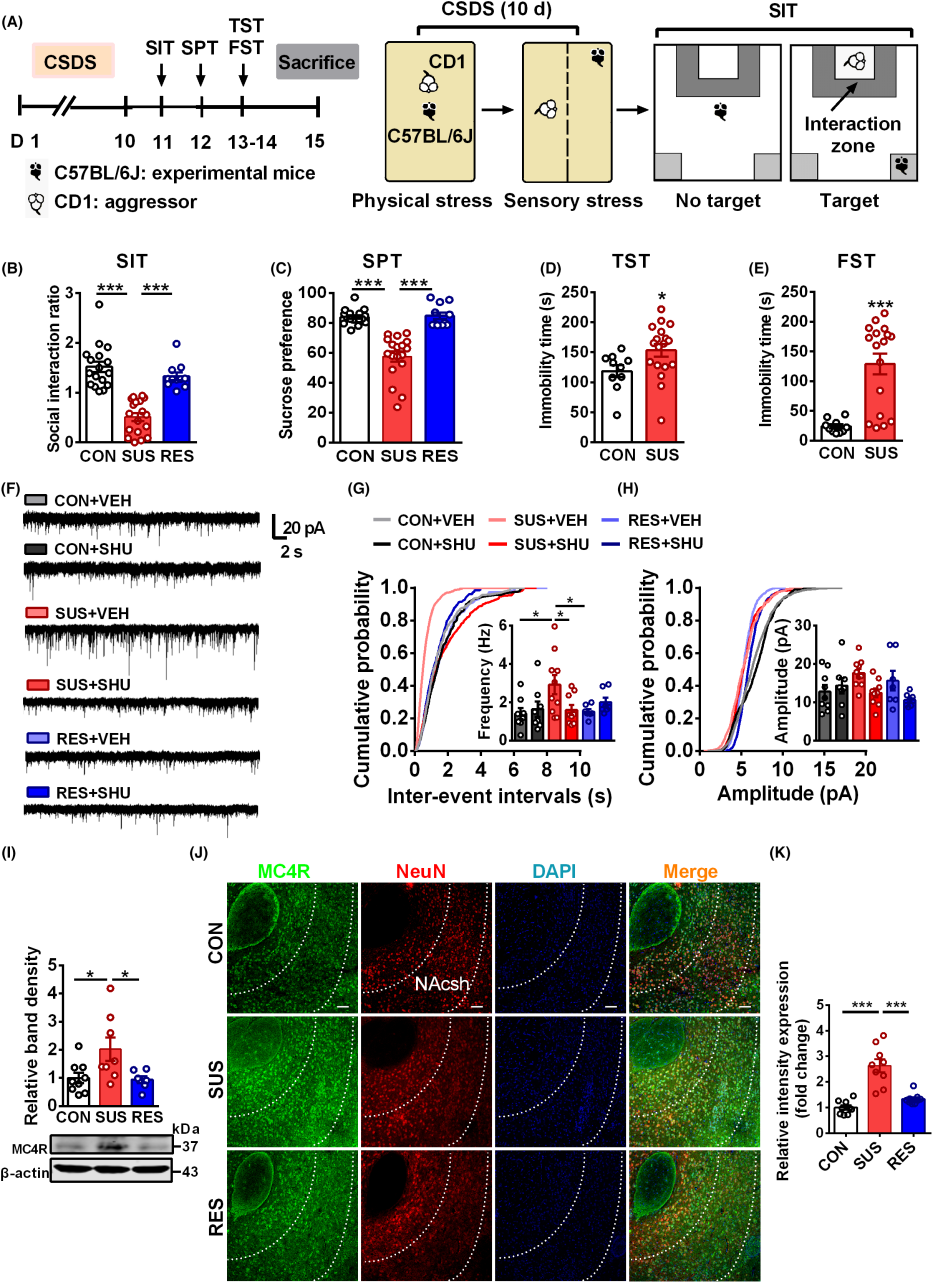
CSDS increases excitatory synaptic transmission in NAcsh through MC4R. (A) Schematic drawing experimental protocol for CSDS. Mice were subjected to consecutive 10‐day social defeat stress and after that behavioral test was performed. (B–E) The susceptible mice that suffered from CSDS displayed depressive‐like behaviors, including decreased social interaction ratio in SIT (B), anhedonia in SPT (C) compared with that of control or resilient mice (*n* = 17, 18, 11), and increased immobility time in TST (D) and FST (E) compared with that of control mice (*n* = 10, 18). (F) Representative AMPAR‐mediated mEPSCs recordings in NAcsh from CON + VEH, CON + SHU, SUS + VEH, SUS + SHU, RES + VEH, RES + SHU. (G) Cumulative graph and representative statistics of mEPSCs frequency showing that MC4R nonselective antagonist (SHU9119, 50 μM) reversed the increased mEPSCs frequency in susceptible mice. (H) Cumulative graph and representative statistics of mEPSCs amplitude showing that SHU9119 (50 μM) reversed the increased mEPSCs amplitude in susceptible mice. (*n* = 8–10 cells from 3 mice per group). (I) Western blotting indicates that susceptible mice significantly increased the level of MC4R protein in NAcsh than that of control or resilient mice (*n* = 7–9). (J) Representative co‐expression of MC4R and NeuN in NAcsh from control, susceptible, and resilient mice. Scale bar = 50 μm. (K) Quantitative expressions of MC4R were significantly increased in NAcsh from susceptible mice compared with that of control or resilient mice (*n* = 9 slices from 3 mice). Data are expressed as mean ± SEM, *p < 0.05, **p < 0.01, ***p < 0.001 by one‐way ANOVA (B, C, I, K) two‐way ANOVA (G, H) followed by Bonferroni's post hoc test, Student's *t* test (D, E). The statistical details can be found in Table S1.

To identify whether stress induces excitatory synaptic alterations via MC4R, we first employed CSDS as an animal model to evaluate the effect of chronic stress on excitatory neurotransmission. After exposure to 10‐day social defeat stress, behavioral tests were performed (Figure 1A). As our previous study,^10^, ^33^ susceptible mice displayed decreased social interaction ratio in SIT (Figure 1B), and decreased sucrose preference in SPT (Figure 1C) when compared with that of control and resilient mice. Susceptible mice displayed increased immobility time in TST (Figure 1D) and FST (Figure 1E) than that of the control.

Since central melanocortin signaling has been reported to mediate the enhancement of hippocampal synaptic plasticity,^34^, ^35^ we first asked whether the dysfunction of excitatory neurotransmission could be observed in susceptible mice. AMPAR‐mediated mEPSCs were recorded by whole‐cell patch‐clamp technique in NAcsh. It was shown that the mEPSCs frequency was increased in NAcsh of susceptible mice than that of control or resilient mice (Figure 1F,G), without change of the amplitude (Figure 1H), indicating increased mEPSCs frequency in NAcsh of susceptible mice. We next investigated the effect of MC4R signaling on CSDS‐induced excitatory neurotransmission. The proper time for α‐MSH is 2–3 h incubation before mEPSCs detection.^4^ It was found that after incubation of SHU9119 (50 μM) for 2 h to antagonize MC4R, the increase in the mEPSCs frequency was blocked in NAcsh of susceptible mice (Figure 1F–H). Meanwhile, the western blotting analysis showed that susceptible mice significantly increased the expression of MC4R in NAcsh compared with that of control and resilient mice (Figure 1I). Immunofluorescent analysis was performed and it was found that MC4R were highly expressed in NeuN, a marker of neuron (Figure S1A), and the expression of MC4R in NAcsh was increased in susceptible mice compared with that of control or resilient mice (Figure 1J,K). These results indicate that CSDS increases MC4R expression and excitatory synaptic transmission in NAcsh.

### 3.2 CSDS activates PVT glutamatergic neurons projection onto NAcsh

**FIGURE 2:**
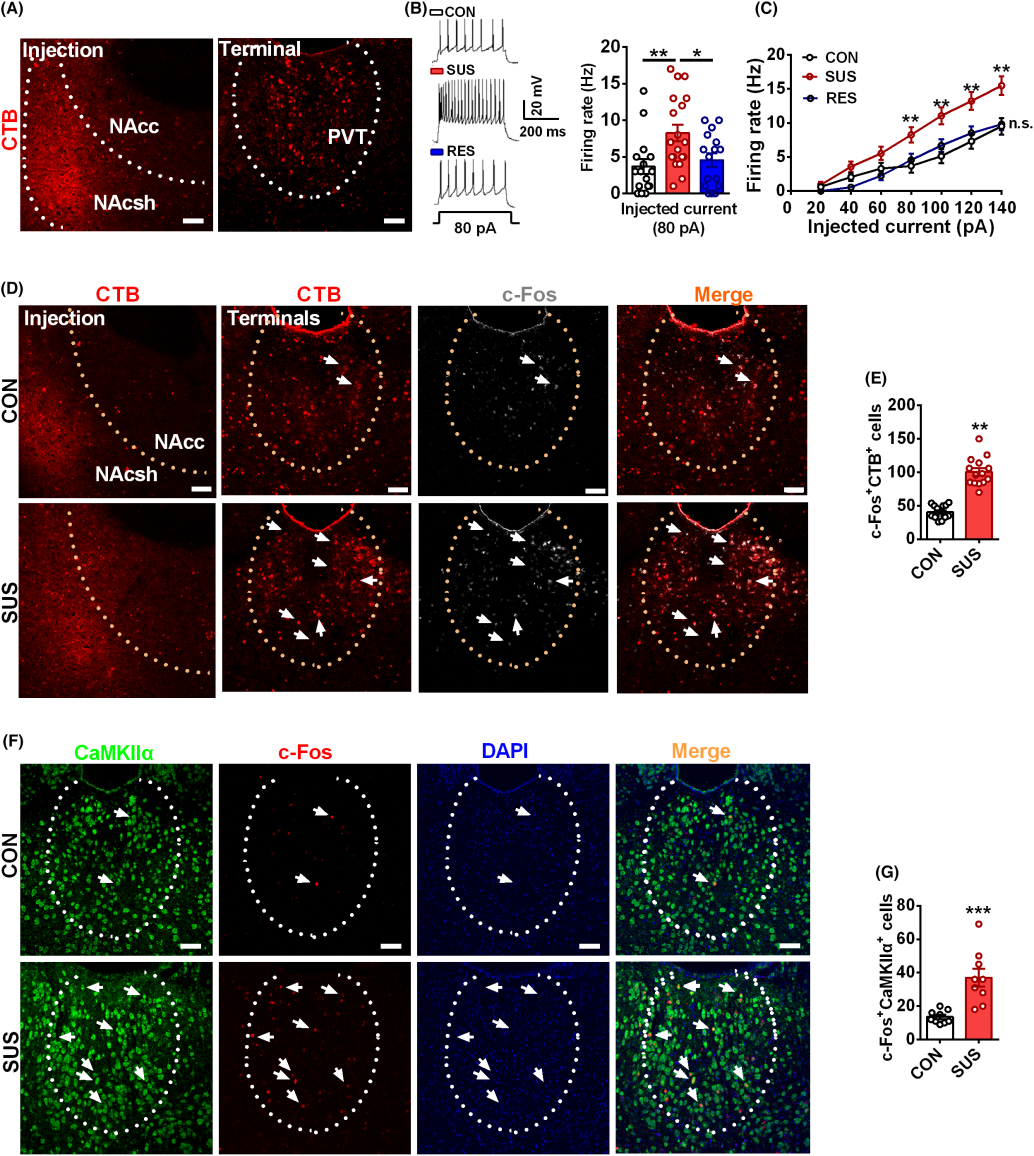
CSDS activates PVT glutamatergic neurons projecting onto NAcsh. (A) Retrograde tracer of CTB showing that NAcsh received synaptic input from PVT, and the injection site in NAcsh (left), the terminal site in PVT (right). Scale bar = 50 μm. (B) Raw traces and statistics of action potential in PVT‐projecting neurons with an infusion of CTB in NAcsh. (C) The summarized data in recorded PVT‐projecting neurons show that susceptible mice significantly increased the firing rate compared with that of control or resilient mice, beginning with a current injection of 80 pA (*n* = 16–19 cells from 3–4 mice). (D) Representative co‐expression of CTB (red) and c‐Fos (white) in the PVT from control and susceptible mice, and the injection site of CTB in NAcsh, the terminal site in PVT. Scale bar = 50 μm. (E) Quantitative co‐expressions of c‐Fos^+^ and CTB^+^ were significantly increased in PVT from susceptible mice compared with that of control (*n* = 15 slices from 5 mice). (F) Representative images showing colocalization of CaMKIIα (green) neurons, c‐Fos (red), and DAPI (blue) in PVT. Scale bar = 50 μm. (G) Co‐expressions of c‐Fos^+^ and CaMKIIα^+^ neurons were increased in PVT from susceptible mice compared with that of control (*n* = 9 slices from 3 mice). Data are expressed as mean ± SEM, *p < 0.05, **p < 0.01, ***p < 0.001 by one‐way ANOVA (B, C) Bonferroni's post hoc test, Student's *t* test (E, G). The statistical details can be found in Table S1.

To explore the related neural projection that led to increased excitatory synaptic input in NAcsh, we stereotaxically injected retrograde tracer cholera toxin B (CTB) conjugated with Alexa Fluor into NAcsh. Strong retrograde tracer signals were detected in PVT (Figure 2A), indicating NAcsh received projections from PVT.

Paraventricular thalamus has been considered a critical thalamic area in regulating energy homeostasis.^17^, ^18^ PVT neurons have also been revealed to be activated upon acute and chronic stress.^22^ To investigate whether PVT is involved in stress susceptibility, we examined the level of c‐Fos in PVT from susceptible mice. It was shown that susceptible mice displayed increased neuronal activity in PVT (Figure S2A,B). Furthermore, the immunofluorescent labels were performed in visualized PVT‐projecting neurons with NAcsh infusion of CTB. Combined with an infusion of CTB into NAcsh, the electrophysiological analysis showed that the firing rate in PVT‐projecting neurons at an injection current of 80 pA was significantly increased in susceptible mice than that in control and resilient mice (Figure 2B,C). Meanwhile, we found an increase in colocalization of c‐Fos and CTB signals in PVT of susceptible mice compared with that of control mice (Figure 2D,E), suggesting that CSDS activates PVT‐projecting neurons onto NAcsh.

Paraventricular thalamus neurons have been demonstrated to provide a primary glutamatergic input into NAc^36^, ^37^ and mediate aversive memory and wakefulness,^14^, ^25^ which are closely related to chronic stress. Accordingly, we measured the activation of glutamatergic neurons in PVT from susceptible mice. An immunofluorescent assay was used to label glutamatergic neurons and GAD67‐expressing neurons. Similarly, the levels of c‐Fos in CaMKIIα‐expressing neurons were significantly increased in susceptible mice (Figure 2F,G), but the level of c‐Fos in GAD67‐expressing neurons was no change (Figure S3A,B). Given the concurrent changes in enhanced activity of glutamatergic neurons in the PVT from susceptible mice, these results demonstrate that CSDS activates the majority of glutamatergic neurons in PVT.

### 3.3 Activation of glutamatergic projection from PVT to NAcsh increases MC4R‐mediated stress susceptibility

**FIGURE 3:**
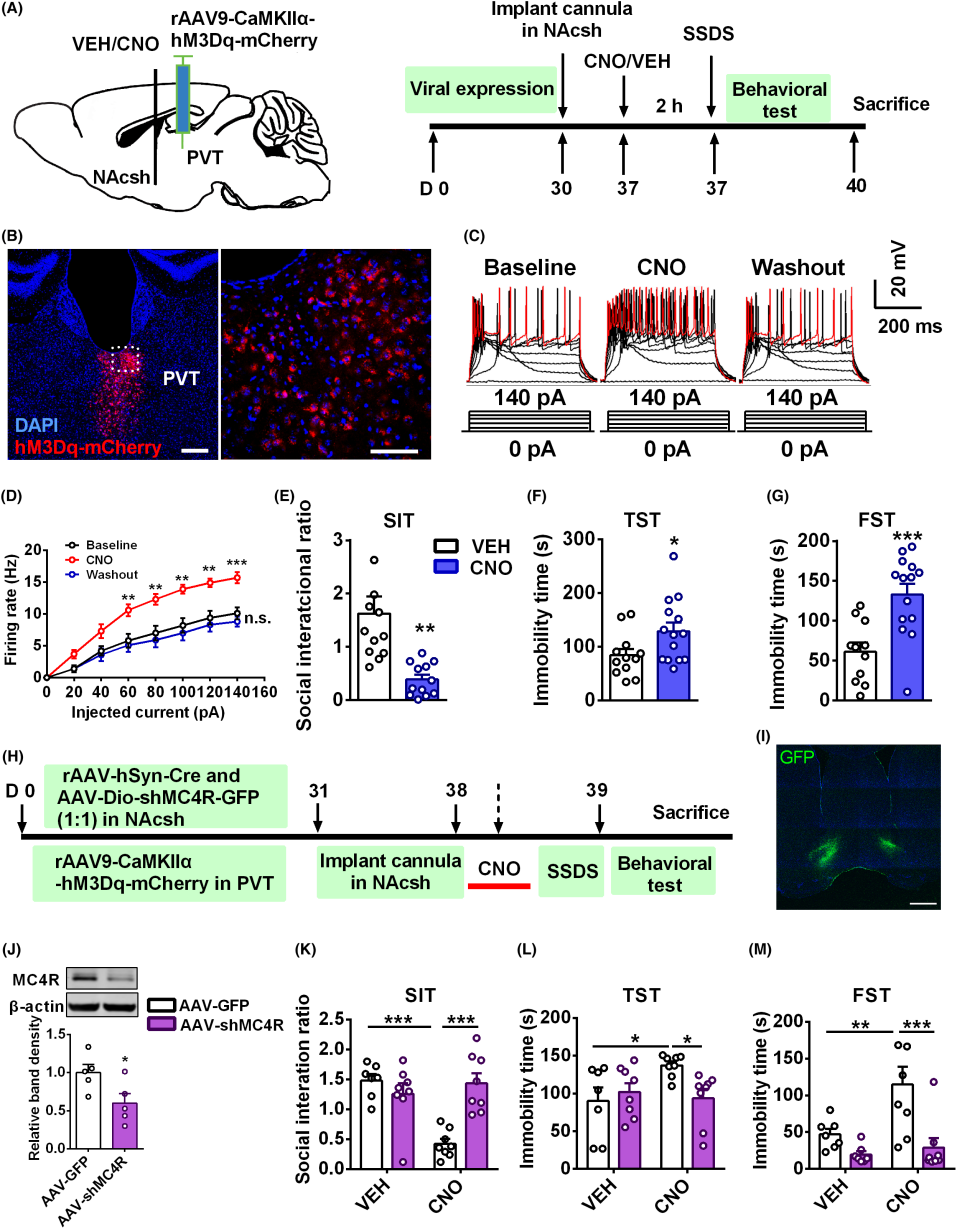
Activation of glutamatergic projection from PVT to NAcsh increases MC4R‐mediated stress susceptibility. (A) Schematic drawing experimental protocol for chemogenetic activation of PVT glutamatergic input to NAcsh before SSDS, single CNO (5 μM) injection into NAcsh of mice that expressed rAAV9‐CaMKIIα‐hM3Dq‐mCherry in the PVT neurons. (B) Representative images showing expression of hM3Dq‐mCherry (red) and DAPI (blue) in PVT neurons. Scale bar = 50 μm. (C) Representative action potential traces recorded in PVT neurons before, during, and after CNO infusion. Raw data show the voltage change by a series of 500 ms current pulses from 0 to 140 pA in 20 pA steps. (D) The firing rate in NAcsh was significantly increased during CNO perfusion (5 μM) of CaMKIIα‐hM3Dq‐mCherry expressed mice compared with that of baseline (*n* = 10 cells from five mice per group). (E–G) Activation of PVT‐NAcsh circuit by CNO shows decreased social interaction ratio in SIT (E), increased immobility time in TST (F), and FST (G) compared with that of vehicle group (*n* = 11–14). (H) Schematic timeline for (I–M). (I) Representative images show the expression of viral cocktail of rAAV‐hSyn‐Cre and AAV‐Dio‐shMC4R‐GFP (1:1) in NAcsh. Scale bar = 50 μm. (J) Western blotting indicates that AAV‐shMC4R significantly reduced the level of MC4R protein in NAcsh than that of AAV‐GFP (*n* = 5). (K) Knockdown of MC4R in NAcsh neurons prevented social avoidance of SSDS‐treated mice from activation of the projection by CNO. (L, M) Knockdown of MC4R decreased immobility time in TST and FST from CNO‐treated mice (*n* = 8–10). Data are expressed as mean ± SEM, *p < 0.05, **p < 0.01, ***p < 0.001 by one‐way ANOVA (D) and two‐way ANOVA (K–M) followed by Bonferroni's post hoc test, Student's *t* test (E–G, J). The statistical details can be found in Table S1.

To elucidate the effect of glutamatergic transmission from PVT to the NAcsh on stress‐induced behavioral consequences, we then asked whether activation of the excitatory synaptic input is sufficient to mimic CSDS‐induced depressive‐like behavior. We injected an excitatory designer receptor exclusively activated by a designer drug (DREADD,^32^ rAAV9‐CaMKIIα‐hM3Dq‐mCherry) into PVT glutamatergic neurons. After 4 weeks, the mice were implanted bilaterally with a metal cannula into NAcsh and recovered for 1 week. Then, clozapine‐N‐oxide (CNO) was injected into NAcsh through the cannula to activate PVT^Glu^‐NAcsh projection 2 h before performing SSDS and behavioral tests (Figure 3A). Fluorescent labeling showed the viral expression in PVT (Figure 3B) and anterograde CaMKIIα‐hM3Dq‐mCherry in the NAcsh (Figure S4A). Electrophysiological recording of the PVT slice confirmed that CNO augmented the firing rate in mCherry‐expressing PVT neurons compared with that of baseline, leading to an increased firing rate under the current step injection (Figure 3C,D). SSDS‐treated mice were housed singly and evaluated 24 h later for behavioral tests. Activation of the PVT‐NAcsh circuit by CNO significantly decreased the social interaction ratio in SIT from 1.625 ± 0.324 to 0.390 ± 0.088 (Figure 3E) and increased immobility time in TST and FST of SSDS‐treated mice (Figure 3F,G), but locomotor activity remained unchanged (Figure S4B,C).

We next examined whether MC4R signaling regulated the modulation of NAcsh projection from PVT glutamatergic neurons in susceptible mice. We found that infusion with SHU9119 (50 μM) in NAcsh abrogated social avoidance induced by CNO of SSDS‐treated mice in SIT (Figure S5A,B). The behavioral test showed that activation of PVT^Glu^‐NAcsh projection failed to increase the immobility time in TST (Figure S5C) and FST (Figure S5D) of SSDS‐treated mice after administration with SHU9119 in NAcsh. To verify the specific effect of MC4R on the projection, we injected a viral cocktail of rAAV‐hSyn‐Cre and AAV‐Dio‐shMC4R‐GFP to selectively knockdown expression of MC4R in NAcsh neurons (Figure 3H–J). Combined with DREADD, behavioral tests displayed that knockdown of MC4R abolished social avoidance of SSDS‐treated mice induced by activation of this projection in SIT (Figure 3K) and decreased the immobility time in TST (Figure 3L) and FST (Figure 3M). Thus, these results suggest that activation of PVT glutamatergic input to NAcsh contributes to stress susceptibility, and MC4R signaling in NAcsh is involved in this process.

### 3.4 Inhibition of PVT glutamatergic projection onto NAcsh ameliorates chronic stress‐induced depressive‐like behavior

**FIGURE 4:**
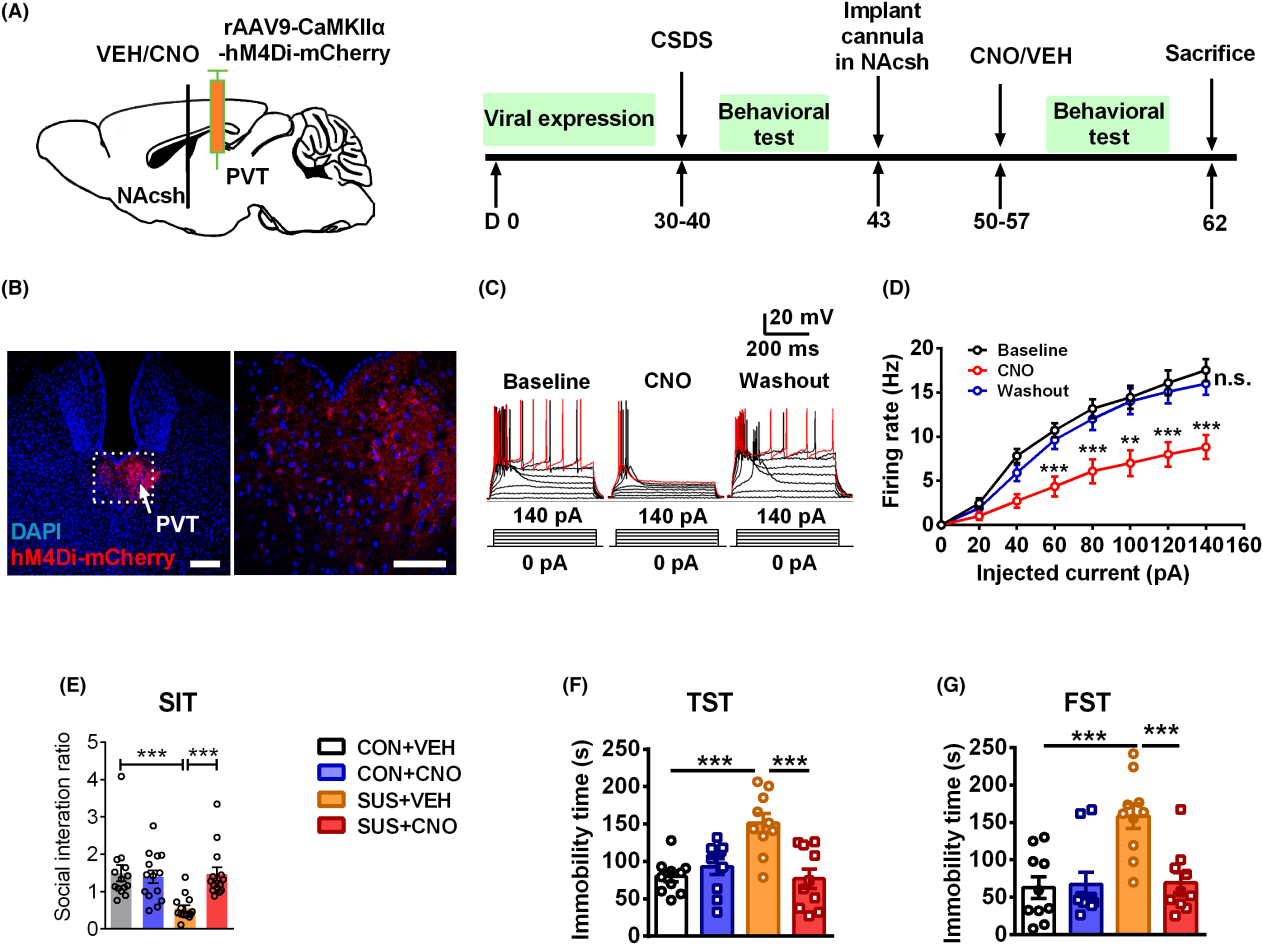
Inhibition of PVT glutamatergic input onto NAcsh ameliorates chronic stress‐induced depressive‐like behavior. (A) Schematic drawing of experimental protocol for chemogenetic inhibition of PVT glutamatergic input to NAcsh after CSDS, injection with CNO into NAcsh of mice that expressed rAAV9‐CaMKIIα‐hM4Di‐mCherry in PVT neurons for 7 days. (B) Representative images show the expression of hM4Di‐mCherry (red) and DAPI (blue) in PVT neurons. Scale bar = 50 μm. (C) Representative action potential traces recorded in PVT neurons before, during, and after CNO infusion. Raw data show the voltage change by a series of 500 ms current pulses from 0 to 140 pA in 20 pA steps. (D) The firing rate in NAcsh was significantly inhibited during CNO perfusion of CaMKIIα‐hM4Di‐mCherry expressed mice compared with that of baseline (*n* = 11 cells from 5 mice per group). (E–G) CNO reversed the decreased social interaction ratio of susceptible mice in SIT (E), decreased immobility time in TST (F), and FST (G) compared with that of SUS + VEH group (*n* = 10–14). Data are expressed as mean ± SEM, ***p < 0.001 by one‐way ANOVA (D) and two‐way ANOVA (E, F, G) followed by Bonferroni's post hoc test. The statistical details can be found in Table S1.

We then investigated the effect of chemogenetic inhibition of PVT glutamatergic projection onto NAcsh on depressive‐like behavior. We first injected an inhibitory DREADD (rAAV9‐CaMKIIα‐hM4Di‐mCherry) into PVT glutamatergic neurons. After 4 weeks of viral expression, the control and susceptible mice were implanted bilaterally with a metal cannula into NAcsh and recovered for 1 week. Then, CNO was injected into NAcsh to inhibit PVT^Glu^‐NAcsh projection before performing a behavioral test (Figure 4A). The virus was effectively expressed in PVT (Figure 4B) and the anterograde terminals in NAcsh (Figure S4D). Electrophysiological recording of action potential in PVT slices confirmed that CNO inhibited the activity in mCherry‐expressing PVT neurons compared with that of baseline, leading to the elimination of the firing rate under the current step injection (Figure 4C,D). We found that injection of CNO into NAcsh in susceptible mice for 1 day or 3 days had no significant effect on the social interaction ratio in SIT (Figure S6A–C). But the repeated injection of CNO into NAcsh in susceptible mice over consecutive 7 days significantly increased the social interaction ratio from 0.549 ± 0.087 to 1.469 ± 0.182 in SIT compared with that of the SUS + VEH group (Figure 4E). Furthermore, inhibition of this projection by CNO decreased immobility time in TST and FST from susceptible mice compared with that of the SUS + VEH group (Figure 4F,G), but locomotor activity remained unchanged (Figure S4E,F). These results indicate that repeated inhibition of glutamatergic neurotransmission from PVT to NAcsh ameliorates CSDS‐induced depressive‐like behavior.

**FIGURE 5:**
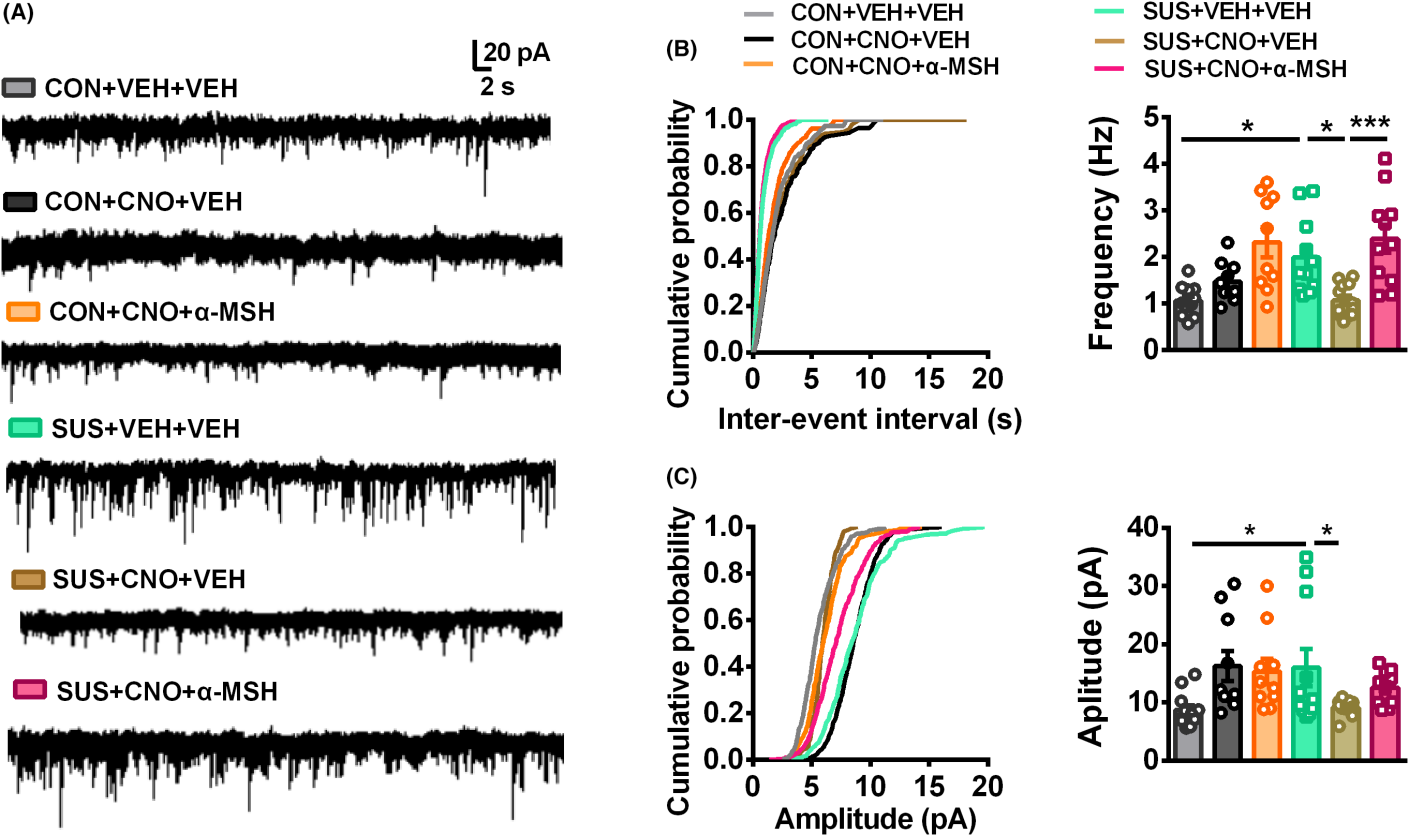
Activation of MC4R signaling abolishes the decreased excitatory neurotransmission by inhibition of PVT glutamatergic input onto NAcsh. (A) Representative AMPAR‐mediated mEPSCs recordings in NAcsh from PVT‐projecting glutamatergic neurons. The group was CON + VEH + VEH, CON + CNO + VEH, CON + CNO + α‐MSH, SUS + VEH + VEH, SUS + CNO + VEH, and SUS + CNO + α‐MSH. (B) Cumulative graphs and statistics of mEPSCs frequency showing that CNO significantly decreased frequency from susceptible mice, but incubation with endogenous MC4R agonist α‐MSH (1 μM) augmented mEPSCs frequency of CNO‐treated mice. (C) Cumulative graph and statistics of mEPSCs amplitude show that CNO significantly decreased amplitude from susceptible mice (*n* = 10–12 cells from 5 to 6 mice per group). Data are expressed as mean ± SEM, *p < 0.05, ***p < 0.001 by two‐way ANOVA (B, C) followed by Bonferroni's post hoc test. The statistical details can be found in Table S1.

Additionally, we further applied MC4R agonist α‐MSH (1 μM) to confirm the molecular mechanism of this circuit. Whole‐cell patch‐clamp recordings displayed that the elevated mEPSCs frequency and amplitude in NAcsh of susceptible mice were reversed by CNO (Figure 5A,B). While incubation of α‐MSH prevented the effect of this chemogenetic inhibition on susceptible mice and restored the elevated mEPSCs frequency in NAcsh of inhibition in this project (Figure 5A,C). Therefore, the above results indicate that MC4R signaling underlies the enhanced excitatory neurotransmission in NAcsh originating from PVT glutamatergic neurons.

## 4 DISCUSSION

In this study, we presented evidence that the enhanced excitatory synaptic transmission in NAcsh was projected from PVT glutamatergic neurons, which were dependent on the melanocortin system, eventually facilitated depressive‐like behavior in response to chronic stress. Our results showed that MC4R strengthened excitatory synaptic transmission in NAcsh from susceptible mice. This enhanced excitatory synaptic input in NAcsh originated from PVT glutamatergic neurons. Chemogenetic activation of PVT glutamatergic projection onto NAcsh enhanced excitatory neurotransmission in the NAcsh and promoted susceptibility to stress. Conversely, Chemogenetic inhibition of PVT glutamatergic projection onto NAcsh reversed the elevated excitatory neurotransmission in the NAcsh and ameliorated depressive‐like behavior induced by CSDS. Furthermore, MC4R signaling in NAcsh participated in this circuit‐mediated stress susceptibility. Our results further provided evidence for the role of a thalamic circuit in stress‐induced depression.

Our previous studies indicated that excitatory synaptic transmission played a key role in chronic stress‐induced depressive‐like behavior.^9^, ^10^ Moreover, NAc conveys stress‐related information to regulate emotional behavior^38^ through altering excitatory synaptic transmission.^39^, ^40^, ^41^, ^42^, ^43^ Stress‐induced dysfunction in forebrain excitatory transmission has led to the development of excitatory synaptic transmission hypothesis of depression.^39^, ^44^, ^45^ NAcsh, as its subregion, is gradually found to play an important role in stress‐induced depressive‐like behavior. Reduced activity of cholinergic interneurons of NAcsh through hyperpolarization‐activated cyclic nucleotide‐gated channel 2 leads to stress‐induced depression.^46^ Chronic stress inhibits A‐type K^+^ channel activity in NAcsh through a glycogen‐synthase kinase 3β‐dependent synaptic plasticity.^47^ Our results show that CSDS increases excitatory synaptic transmission in NAcsh, contributing to chronic stress‐induced depressive‐like behavior. Thus, synaptic dysfunction of NAcsh may account for stress susceptibility. Moreover, neurovascular coupling and hemodynamic responses have also been identified to be essential in the impact of excitatory neurotransmission in the brain.^48^, ^49^ CSDS induces neurovascular pathology of NAc, which promotes depressive‐like behavior.^50^ Future study needs to investigate whether the neurovascular pathology is involved in the dysfunction of excitatory neurotransmission in NAcsh induced by CSDS.

MC4R signaling is essential in mediating synaptic plasticity. The strength of excitatory synapses has been affected by exposure to chronic stress, due to the activation of MC4Rs in NAc.^4^ For example, administration of MC4R agonist increases long‐term potentiation in the hippocampus of mice.^34^ Here, we demonstrate that activation of MC4R results in the enhanced frequency and amplitude of mEPSCs in NAcsh. These suggest that not only do PVT‐projecting neurons provide excitatory synaptic input into NAcsh, but also MC4R in NAcsh may act on excitatory synaptic strength. Further study is needed to explore the changes in synaptic strengths of this projection. Emerging evidence reveals the association of MC4R signaling with emotional behavior. And activation of MC4R signaling contributes to anxiety and depressive‐like behavior.^3^, ^51^ The stimulatory effects of α‐MSH are thought to be mainly mediated by MC4R. Even α‐MSH and γ‐MSH also display affinity to MC3R, whose main function is involved in the central regulation of feeding.^52^, ^53^, ^54^ We present here that susceptible mice displayed an increased expression of MC4R in NAcsh. Although MC4R has also been identified to contribute to sex difference in energy metabolism. For example, the increased food intake and body weight induced by MC4R deficiency is more obvious in males than that in females.^55^ Considering the limitation of the CSDS model used in our present work, which is confined only to male mice, the effect of MC4R deficiency on behavioral alteration could not be determined in female mice. Thus, our data could not exclude the possibilities of sex‐difference in stress susceptibility related to MC4R signaling. Actually, sex‐specific behavior in reward and punishment processing has also been displayed in dopamine metabolism in the striatum.^56^ Further study with other animal models^57^ can be used to investigate whether MC4R mediates the sex difference in stress‐related negative emotional behaviors.

Paraventricular thalamus, as a well‐known “way‐station” that assembles information and regulates stress‐related mesolimbic reward systems, might also play a key role in emotional behaviors.^23^, ^58^, ^59^ Our experiments showed that CSDS increased the activity of PVT‐glutamatergic projection onto NAcsh. This is consistent with previous studies, which provide potential clues that the activity of PVT neurons may be associated with stress and emotional behaviors.^20^, ^23^, ^58^ and that excitatory synaptic adaptation in NAcsh is involved in stress‐induced depressive‐like behavior.^39^, ^40^, ^41^, ^47^ Importantly, chronic fluoxetine treatment reduces the activity in PVT of depressive mice^28^ and human neuroimaging studies identify abnormal neural connections in PVT of major depressive patients.^22^ Therefore, it is essential to investigate PVT neural projections that facilitate depressive‐like behavior. By using chemogenetic approaches, we showed that repeated inhibition of the PVT glutamatergic input to NAcsh ameliorated CSDS‐induced depressive‐like behavior. Conversely, acute chemogenetic activation of this projection increased the susceptibility to SSDS. These suggest that the glutamatergic synaptic input from PVT to NAcsh contributes to stress susceptibility induced by social stress.

It has been reported that central circuits, including PVT projection to the NAc, are involved in both metabolic homeostasis and emotional status.^18^, ^22^, ^60^, ^61^ Meanwhile, MC4R signaling acts an important role in energy expenditure and stress‐related behavior.^19^, ^51^, ^62^ Additionally, our results showed that MC4R signaling strengthened excitatory neurotransmission in NAcsh, and that neurons in NAcsh received dense glutamatergic input from PVT, pointing to a PVT‐NAc circuit might be dependent on MC4R signaling. Accordingly, we found that inhibition of MC4R signaling reversed depressive‐like behavior, which was induced by chemogenetic activation of PVT‐glutamatergic input to NAcsh. Previous studies suggested that α‐MSH could be released from axon terminals,^63^ and function on excitatory postsynaptic plasticity to facilitate stress‐related behavior.^4^ Direct stimulation of MC4R via α‐MSH abolished the decreased frequency and amplitude induced by chemogenetic inhibition on this projection of CSDS‐treated mice. Our results indicate that MC4R signaling in NAcsh strengthens the excitatory synaptic transmission contributing to depressive‐like behavior, and synaptic input from PVT glutamatergic neurons is involved in this process.

Our study documents, for the first time, that CSDS induces the reorganization of excitatory synaptic transmission from PVT to the NAcsh circuit, which is dependent on the melanocortin system, resulting in stress susceptibility. These results raise the possibility of metabolic underpinnings to protect against corresponding stress‐induced depressive‐like behavior.

## AUTHOR CONTRIBUTIONS

Q.D. and S.Q.Z. wrote the manuscript and performed most experiments. P.F.Y. took part in constructing CSDS models. W.‐T.D. and J.‐L.W. took part in the behavioral tests and analysis. J.‐G.C. and F.W. designed the project and revised the manuscript. L.‐H.L. conceived the project and designed the experiments.

## CONFLICT OF INTEREST

The authors declare that they have no conflict of interest.

## Supporting information


Appendix S1.

Figure S1.

Figure S2.

Figure S3.

Figure S4.

Figure S5.

Figure S6.

## Data Availability

The data that support the findings of this study are available from the corresponding author upon reasonable request.
