# Supplementary material for: A thalamic circuit facilitates stress susceptibility via melanocortin 4 receptor‐mediated activation of nucleus accumbens shell
Source: CNS Neurosci Ther. 2022 Dec 12;29(2):646–58. doi: 10.1111/cns.14046 (PMC9873525; doi:10.1111/cns.14046)
Supplement: Supplementary file 1 — Appendix S1. [file CNS-29-646-s006.docx]

**Supporting Information**

**A thalamic circuit facilitates stress susceptibility via melanocortin 4 receptor-mediated activation of nucleus accumbens shel**

Qiao Deng^1,#^, Shao-Qi Zhang^1,#^, Ping-Fen Yang^1^, Wan-Ting Dong^1^, Jia-Lin Wang^1^, Jian-Guo Chen^1,2,3,4,5^, Fang Wang^1,2,3,4,5^ and Li-Hong Long^1,2,3,*^

^1^Department of Pharmacology, Tongji Medical College, Huazhong University of Science and Technology, Wuhan City, Hubei 430030, China;

^2^The Research Center for Depression, Tongji Medical College, Huazhong University of Science and Technology, 430030, Wuhan, China;

^3^The Key Laboratory for Drug Target Researches and Pharmacodynamic Evaluation of Hubei Province, 430030, Wuhan, China;

^4^Key Laboratory of Neurological Diseases (HUST), Ministry of education of China, Wuhan, Wuhan City, Hubei 430030, China;

^5^Laboratory of Neuropsychiatric Diseases, The Institute of Brain Research, Huazhong University of Science and Technology, 430030, Wuhan, China.

^#^Equally contributed authors

*Correspondence to: Dr. L.-H. Long, Department of Pharmacology, Tongji Medical College, Huazhong University of Science and Technology, 13 Hangkong Road, Wuhan, Hubei, China 430030. E-mails: longlihong@hust.edu.cn

Tel: +86-27-83692636; FAX: +86-27-83692608

**Supplemental methods**

**Behavioral assessments**

After social defeat, mice were housed singly and evaluated 24 h later for social interaction test (SIT). SIT was performed according to previous describes.^1^ All behavioral assessments were recorded by ANY-maze video-tracking software (Stoelting Co. New jersey, USA). In the first stage (No target), the experimental mice were placed in an open-filed arena (45 × 45 × 45 cm^3^) containing an empty mesh cage (10 × 6 × 8 cm^3^). In the second stage (Target), a novel aggressive CD1 mouse was placed in the mesh cage, and experimental mice were reintroduced into the arena. Time spent in the area of interaction zone (14 × 26 cm) and in the corner zone (10 × 10 cm) were measured individually. The interaction ratio was calculated as (the time spent in the interaction zone in the second test “Target”) versus (the time spent in the interaction zone in the first test “No target”). Mice were deemed as susceptible when the ratio was < 1 or control and resilient when the ratio was > 1.

Sucrose preference test (SPT). After CSDS, each mouse was habituated to two 50 ml tubes filled with drinking water and 1% sucrose solution for two consecutive days to eliminate side preference. Then, the mice were given access to a two-bottle choice of water (bottle W) or 1% sucrose solution (bottle S). The position of the two bottles was exchanged at time points of 12 h and weighed at 24 h. Sucrose preference was calculated as the percentage (weight of sucrose consumed (bottle S) versus total weight consumed (bottle W + bottle S) × 100). The details were assessed as previous described.^2^

Tail suspension test (TST) was evaluated according to previous procedure.^3^ the experimental mice were suspended by their tails with tape (1 cm from the tail tip) and roughly 25 cm fixed above the surface. Plastic tubes were placed over the tails to avoid climbing up on to their tail. The animals were video-recorded for 6 min and the immobility time were analyzed.

Forced swim test (FST) was performed 24 h after completion of TST. The experimental mice were habituated at least 1-h in the behavioral room. Mice were placed in a glass cylinder (35 cm in height, 15 cm in diameter) containing 20 cm-depth lukewarm water (25 ± 1 °C). FST session was recorded for 6 min, and the water was replaced in each trial. The immobility time of the last 4 min was assessed.

Locomotor activity. Mice were individually placed in an open field with dim light for 10 min, and locomotor activity was evaluated as total distance and speed traveled. Their paths were recorded by ANY-maze video-tracking software (Stoelting Co. New jersey, USA).

**Western blotting analysis**

Brain tissues were lysed on ice in RIPA lysis buffer (Thermo Scientific, MA, USA) containing protease and phosphatase inhibitors. Protein samples were separated by 10% SDS-PAGE and transferred to nitrocellulose membranes. The transferred membranes were blocked with 5% bovine serum albumin (BSA) in Tris-buffered saline containing 0.1% Tween-20 for 2 h at room temperature and incubated with primary antibodies overnight at 4 °C. The membranes were incubated in horseradish peroxidase secondary antibodies for 2 h at room temperature and kept in a dark place. After that, images were visualized and quantified using an Odyssey Imaging System (LI-COR Biosciences, NEB, USA). The details were followed as previous reports.^4^The information of antibodies was listed as follows: anti-MC4R antibody (1:1000 dilution; Abcam, ab24233, Cambridge, UK), anti-β-actin antibody (1:2000 dilution; Sigma-Aldrich, A1978, MO, USA), Goat anti-rabbit IgG (H+L) secondary antibody (1:10000 dilution; Invitrogen, 31460, Paisley, UK), Goat anti-mouse IgG (H+L) secondary antibody (1:10000 dilution; Invitrogen, 31430, Paisley, UK).

**Immunofluorescence**

Mice were anesthetized with sodium pentobarbital (45 mg/kg) by intraperitoneal injection (*i.p.*), and perfused with 4% paraformaldehyde in phosphate buffer saline (PBS). Brains were sectioned at 30 μm coronal sections using freezing vibratome (CM1900, Leica Microsystems, Wetzlar, Germany). Brain slices were blocked in 3% BSA containing 0.3% TritonX-100 and 2% normal donkey serum for 2 h at room temperature. Then, sections were incubated with the following primary antibodies overnight at 4 °C, anti-c-Fos antibody (1:1000 dilution; Abcam, ab208942, Cambridge, UK), anti-c-Fos antibody (1:1000 dilution; Abcam, ab190289, Cambridge, UK), anti-CTB antibody (1:10000 dilution; List Biological labs, Inc., 703, CA, USA), anti-CaMKIIα antibody (1:200 dilution; Novus biological, NB100-81830, CO, USA), anti-GAD67 antibody (1:50 dilution; Abcam, ab26116, Cambridge, UK). After that, sections were washed in PBS for 10 min followed by the Alexa Fluor-conjugated secondary antibody (1:800 dilution; Invitrogen, A21206, Paisley, UK) for 2 h, Finally, sections were washed in PBS and mounted for imaging through confocal microscope (FV1000, Olympus, Tokyo, Japan).^5^ The number of c-Fos positive cells, CTB and these positive cells were counted with ImageJ software (NIH, MD, USA). Additionally, to verify the viral fluorescence into PVT and NAcsh, similarly, brain slices containing PVT and NAcsh were mounted for imaging.

**Virus injection and cannula implantation**

Alexa Fluor 647-chorela toxin subunit B (CTB) (Invitrogen, Paisley, UK) were used to detect retrograde tracer in NAcsh. Viral cocktail of rAAV-hSyn-Cre and AAV-Dio-shMC4R-GFP (1:1) were purchased from OBiO company (Shanghai, China) and the final titer was 6.0 × 10^12^ GC/ml. rAAV9-CaMKIIα-hM3Dq-mCherry and rAAV9-CaMKIIα-hM4Di-mCherry were purchased from BrainVTA Company (Wuhan, China). The final titer was 6.0 × 10^12^ GC/ml.

For stereotaxic viral injection, CTB (0.2 μl) were bilaterally microinjected into NAcsh (AP = + 1.6 mm; ML = ± 0.8 mm; DV = - 4.7 mm relative to the bregma)^6^ through a micro syringe pump controller (World Precision Instruments, Sarasota, USA) and mice were waited for one week to infection. Viral cocktail of rAAV-hSyn-Cre and AAV-Dio-shMC4R-GFP (1:1) (0.2 μl) were bilaterally microinjected into NAcsh. rAAV9-CaMKIIα-hM3Dq-mCherry or rAAV9-CaMKIIα-hM4Di-mCherry (0.2 μl) were unilaterally microinjected into PVT (AP = - 1.4 mm; ML = - 0.5 mm; DV = - 2.95 mm relative to the bregma, with a 10° angle towards the midline).^7^ These viruses were microinjected with a rate of 0.05 μl /min. Mice were allowed at least 3-4 weeks to recover and to express virus after surgery. Efficiency of viral infection were confirmed by confocal microscopy and western blotting.

For pharmacological experiments. 22-gauge stainless steel guide cannulas (RWD life Science Co., Shenzhen, China) were bilaterally implanted above the NAcsh (AP = + 1.6 mm; ML= ± 0.8 mm; DV = - 4.6 mm) through stereotaxic apparatus for drug infusion 1 week later.

**References**

1. Golden SA, Covington HE, 3rd, Berton O, Russo SJ. A standardized protocol for repeated social defeat stress in mice. *Nat Protoc.* 2011;6(8):1183-1191.

2. Li MX, Zheng HL, Luo Y, et al. Gene deficiency and pharmacological inhibition of caspase-1 confers resilience to chronic social defeat stress via regulating the stability of surface AMPARs. *Mol Psychiatry.* 2018;23(3):556-568.

3. Can A, Dao DT, Terrillion CE, et al. The tail suspension test. *J Vis Exp.* 201259):e3769.

4. He JG, Zhou HY, Xue SG, et al. Transcription Factor TWIST1 Integrates Dendritic Remodeling and Chronic Stress to Promote Depressive-like Behaviors. *Biol Psychiatry.* 2021;89(6):615-626.

5. Shen CJ, Zheng D, Li KX, et al. Cannabinoid CB1 receptors in the amygdalar cholecystokinin glutamatergic afferents to nucleus accumbens modulate depressive-like behavior. *Nat Med.* 2019;25(2):337-349.

6. Cheng J, Umschweif G, Leung J, Sagi Y, Greengard P. HCN2 Channels in Cholinergic Interneurons of Nucleus Accumbens Shell Regulate Depressive Behaviors. *Neuron.* 2019;101(4):662-672 e665.

7. Ren S, Wang Y, Yue F, et al. The paraventricular thalamus is a critical thalamic area for wakefulness. *Science.* 2018;362(6413):429-434.

**Supplemental Figures**


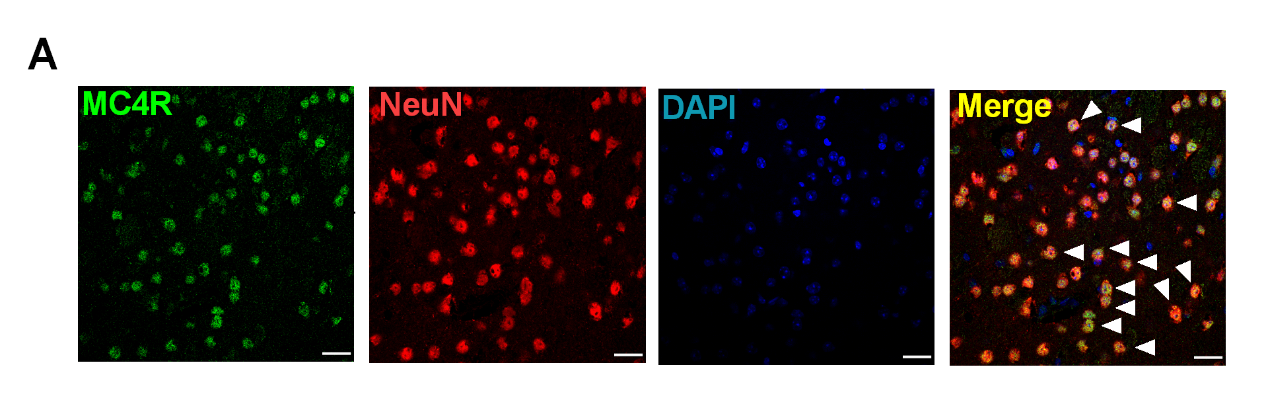


**FIGURE S1** MC4R is expressed in neurons of NAcsh. (A) Representative co-expression of MC4R (green), NeuN (red), and DAPI (blue) in NAcsh. Scale bar = 50 μm. The statistical details can be found in Supplemental Table S1.


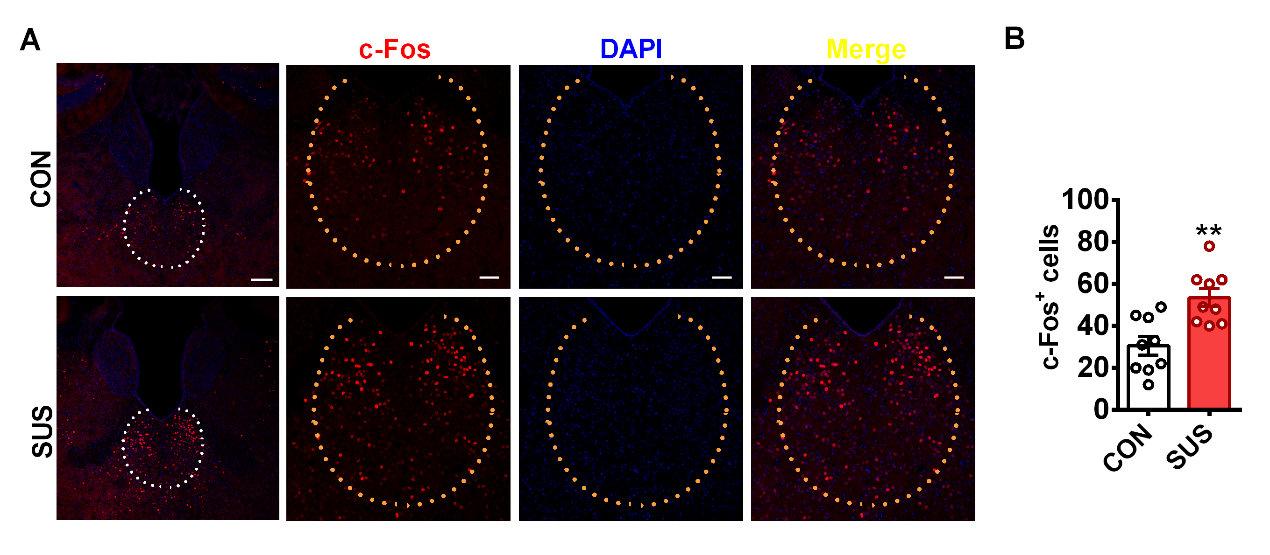


**FIGURE S2** CSDS increases the neuronal activity in PVT. (A) Representative expression of c-Fos^+^ (red) in the PVT from control and susceptible mice. (B) Quantitative expressions of c-Fos^+^ were significantly increased in PVT from susceptible mice compared with that of control (*n* = 9 slices from 3 mice). Scale bar = 50 μm. Data are expressed as mean ± SEM, ***p* < 0.01 followed by Student’s *t*-test (B). The statistical details can be found in Supplemental Table S1.


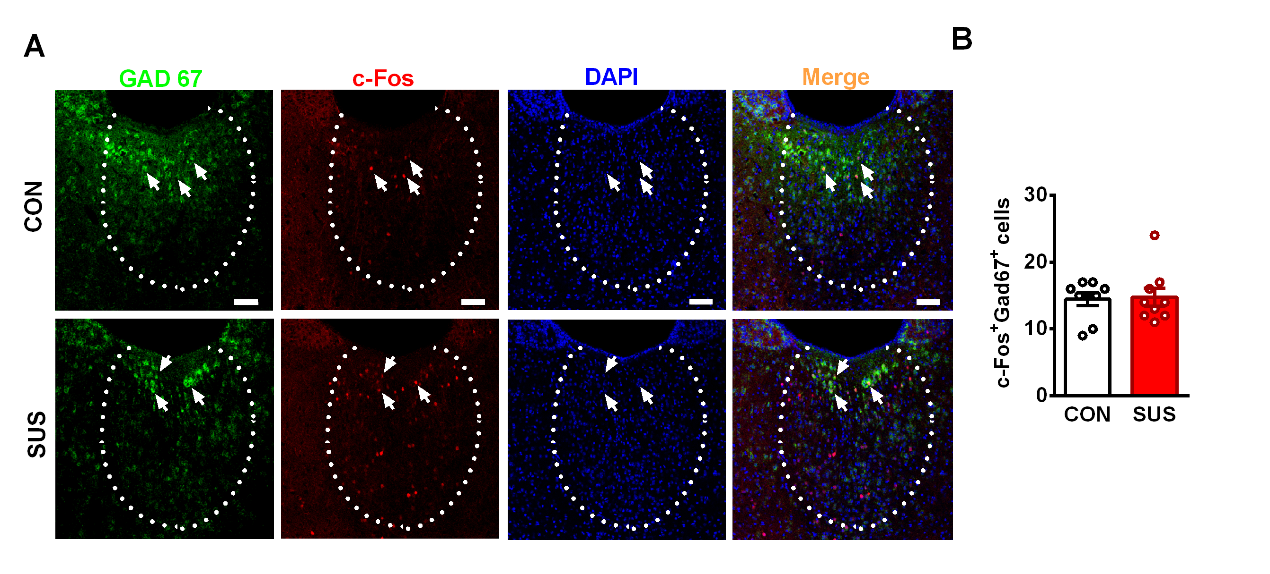


**FIGURE S3** The activity of GAD67-expressing neurons is unaltered by CSDS. (A) Representative co-expression of GAD67^+^ (green), c-Fos^+^ (red), and DAPI (blue) in the PVT from control and susceptible mice. (B) Quantitative co-expressions of c-Fos^+^ and GAD67-expressing neurons were no different in PVT from susceptible mice compared with that of control (*n* = 9 slices from 3 mice). Scale bar = 50 μm. Data are expressed as mean ± SEM, *p* > 0.05 followed by Student’s *t*-test (B). The statistical details can be found in Supplemental Table S1.


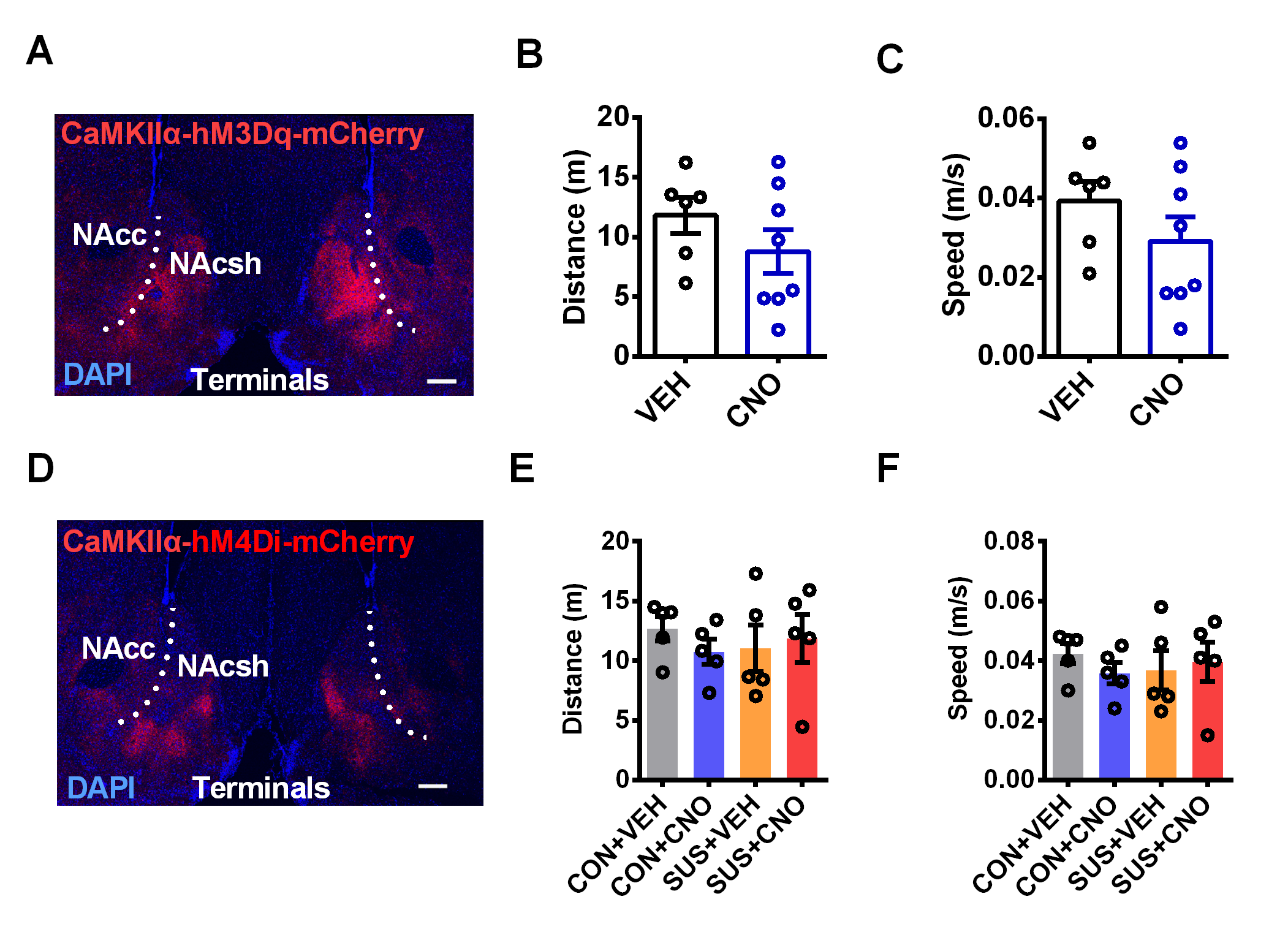


**FIGURE S4** The effect of chemogenetic regulation on PVT^Glu^-NAcsh circuit and its role in locomotor activity. (A) Representative images showing terminals of anterograde CaMKIIα-hM3Dq-mCherry (red) and DAPI (blue) in the NAcsh from PVT glutamatergic neurons. Scale bar = 200 μm. (B, C) Locomotor activity remained unchanged between VEH and CNO group (*n* = 6-8). (D) Representative images showing terminals of anterograde CaMKIIα-hM4Di-mCherry (red) and DAPI (blue) in NAcsh from PVT glutamatergic neurons. Scale bar = 200 μm. (E, F) Locomotor activity displayed unaltered from different groups (*n* = 5). Data are expressed as mean ± SEM, *p* > 0.05 by one-way ANOVA (E, F) followed by Bonferroni’s *post hoc* test, Student’s *t*-test (B, C). The statistical details can be found in Supplemental Table S1.


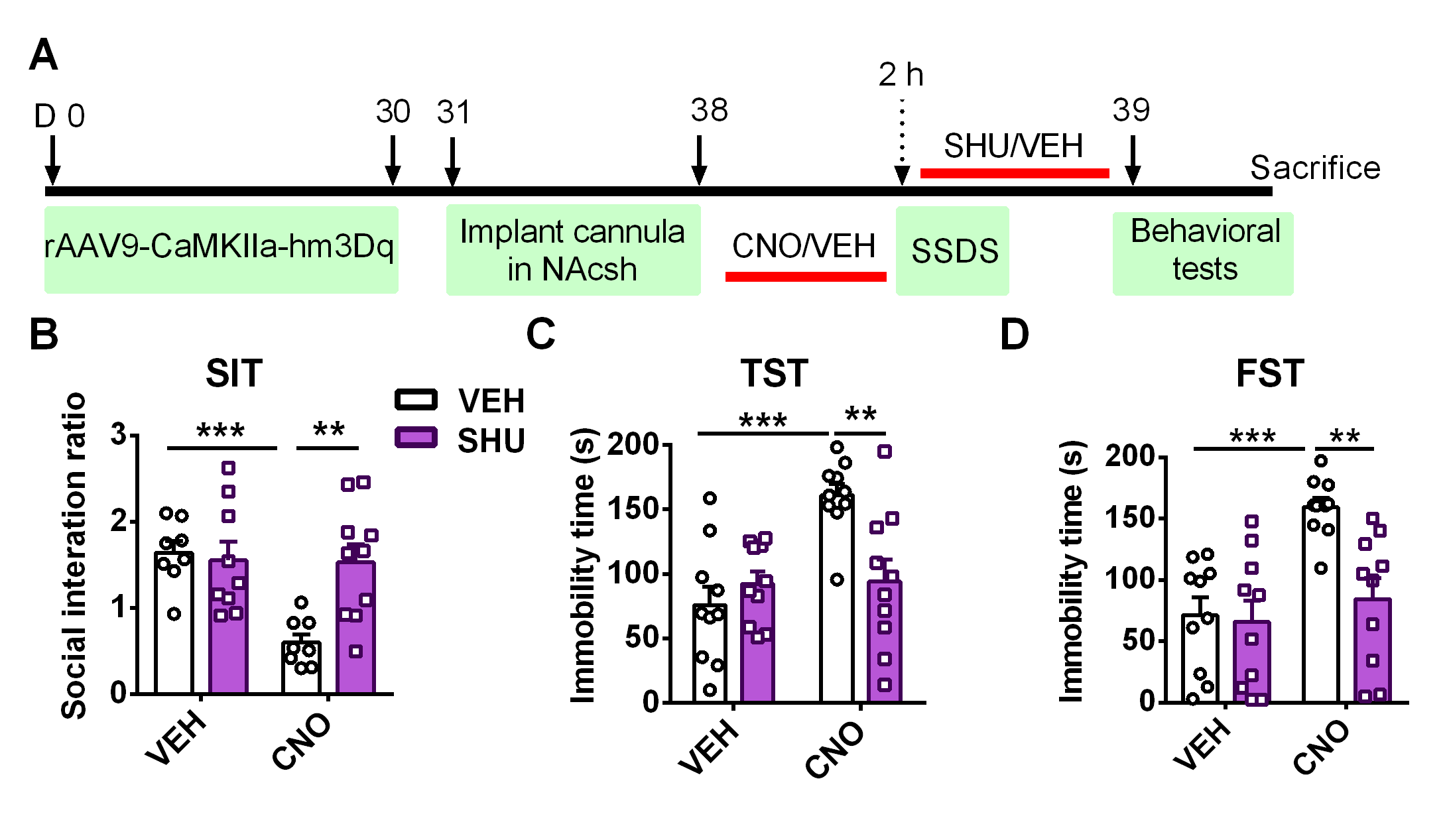


**FIGURE S5** Inhibition of MC4R abolishes the activation of PVT^Glu^-NAcsh circuit induced stress susceptibility. **(A)** Schematic timeline for **(B-D)**. **(B)** Injection of SHU9119 (50 μM) into NAcsh prevented social avoidance of SSDS-treated mice from activation of the projection by CNO. **(C, D)** SHU91919 decreased immobility time in TST and FST from CNO-treated mice (*n* = 8-10). Data are expressed as mean ± SEM, ***p* < 0.01, ****p* < 0.001 by two-way ANOVA **(B-D)** followed by Bonferroni’s *post hoc* test. The statistical details can be found in Supplemental Table S1.


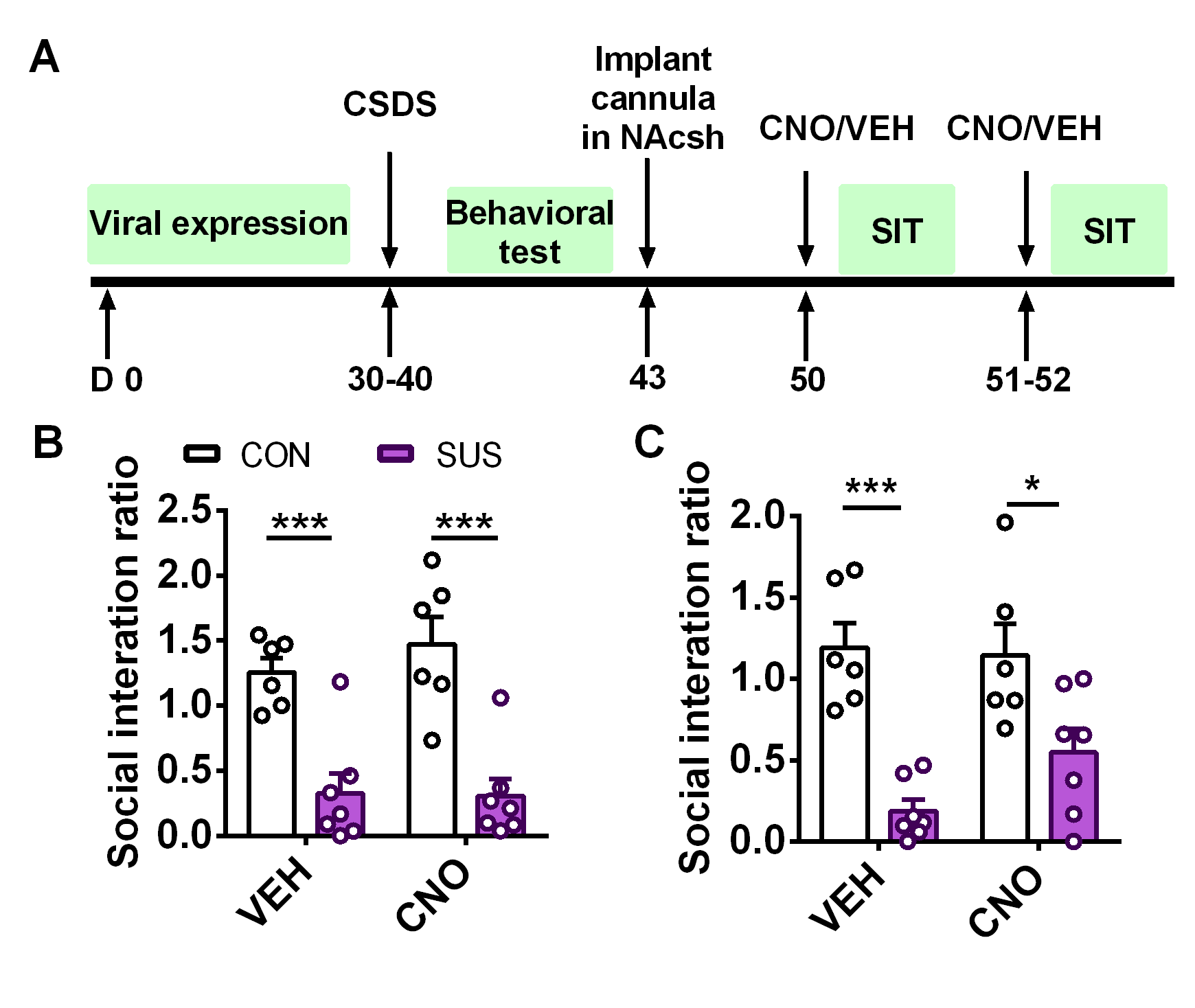


**FIGURE S6** Inhibition of PVT^Glu^-NAcsh circuit for one or three days has no effect on CSDS-induced social avoidance. **(A)** Schematic timeline for **(B-C)**. **(B)** Acute injection of CNO (5 μM) into NAcsh had no effect on social avoidance of susceptible mice. **(C)** Injection of CNO (5 μM) into NAcsh for 3 days had no effect on social avoidance of susceptible mice (*n* = 7-8). Data are expressed as mean ± SEM, **p* < 0.05, ****p* < 0.001 by two-way ANOVA **(B, C)** followed by Bonferroni’s *post hoc* test. The statistical details can be found in Supplemental Table S1.

**Supplemental Table S1. Statistical analysis for Figures 1-5 and Figures S1-6.**

| **Figures and numbers of animals or cells** | **Statistical analysis** | ***Post hoc tests*** | ***mean ± SEM*** |
| --- | --- | --- | --- |
| 1B: SIT  CON (n = 17)  SUS (n = 18)  RES (n = 11) | One-way ANOVA  *F* (2, 43) = 36.870, *p* < 0.001 | Bonferroni's  post hoc test  CON vs SUS, *p* < 0.001  SUS vs RES, *p* < 0.001 | CON 1.518 ± 0.107  SUS 0.512 ± 0.080  RES 1.328 ± 0.077 |
| 1C: SPT  CON (n = 17)  SUS (n = 18)  RES (n = 11) | One-way ANOVA  *F* (2, 43) = 3.592, *p* < 0.001 | Bonferroni's  post hoc test  CON vs SUS, *p* < 0.001  SUS vs RES, *p* < 0.001 | CON 83.940 ± 1.207  SUS 57.510 ± 3.415  RES 84.800 ± 2.256 |
| 1D: TST  CON (n = 10)  SUS (n = 18) | Unpaired *t* test  *t* = 2.154, *p* = 0.041 |  | CON 118.700 ± 10.120  SUS 153.400 ± 10.550 |
| 1E: FST  CON (n = 10)  SUS (n = 17) | Unpaired *t* test  *t* = 4.543, *p* = 0.001 |  | CON 23.830 ± 3.506  SUS 128.900 ± 17.480 |
| 1G: mEPSCs frequency  CON + VEH (n = 8) CON + SHU (n = 8)  SUS + VEH (n = 10)  SUS + SHU (n = 9)  RES + VEH (n = 7)  RES + SHU (n = 7) | Two-way ANOVA  SUS × SHU: *F* (1, 31) = 3.837, *p* = 0.059  SUS: *F* (1, 31) = 1.928, *p* = 0.175  SHU: *F* (1, 31) = 3.493, *p* = 0.071 | Bonferroni's post hoc test CON + VEH vs SUS + VEH, *p* = 0.020  SUS + VEH vs SUS + SHU, *p* = 0.019  SUS + VEH vs RES + VEH, *p* = 0.020 | CON + VEH 1.414 ± 0.287  CON + SHU 1.639 ± 0.401  SUS + VEH 2.923 ± 0.507  SUS + SHU 1.604 ± 0.264  RES + VEH 1.516 ± 0.126  RES + SHU 2.006 ± 0.232 |
| 1H: mEPSCs amplitude  CON + VEH (n = 9)  CON + SHU (n = 8)  SUS + VEH (n = 10)  SUS + SHU (n = 9)  RES + VEH (n = 7)  RES + SHU (n = 7) | Two-way ANOVA  SUS × SHU: *F* (1, 32) = 5.082, *p* = 0.031  SUS: *F* (1, 32) = 1.478, *p* = 0.233  SHU: *F* (1, 32) = 1.004, *p* = 0.324 | Bonferroni's post hoc test CON + VEH vs SUS + VEH, *p* = 0.048  SUS + VEH vs  SUS + SHU, *p* = 0.033  SUS + VEH vs RES + VEH, *p* = 0.684 | CON + VEH 12.790 ± 1.698  CON + SHU  14.350 ± 2.008  SUS + VEH 17.670 ± 1.125  SUS + SHU 12.470 ± 1.149  RES + VEH 15.670 ± 2.596  RES + SHU 10.630 ± 2.674 |
| 1I: Expression of MC4R in NAcsh  CON (n = 9)  SUS (n = 8)  RES (n = 7) | One-way ANOVA  *F* (2, 21) = 4.891, *p* = 0.018 | Bonferroni's  post hoc test  CON vs SUS, *p* = 0.039  SUS vs RES, *p* = 0.040 | CON 1.000 ± 0.184  SUS 2.025 ± 0.416  RES 0.937 ± 0.120 |
| 1K: Fluorescence expression of MC4R in neurons of NAcsh  CON (n = 9 slices from 3 mice)  SUS (n = 9 slices from 3 mice)  RES (n = 9 slices from 3 mice) | One-way ANOVA  *F* (2, 24) = 28.71, *p* < 0.001 | Bonferroni's  post hoc test  CON vs SUS, *p* < 0.001  SUS vs RES, *p* < 0.001 | CON 1.000 ± 0.088  SUS 2.637 ± 0.256  RES 1.335 ± 0.071 |
| 2B: AP-80 pA  CON (n = 16)  SUS (n = 19)  RES (n = 16) | One-way ANOVA  *F* (2, 48) = 5.728, *p* = 0.006 | Bonferroni's  post hoc test  CON vs SUS, *p* = 0.009  SUS vs RES, *p* = 0.043 | CON 3.688 ± 0.990  SUS 8.263 ± 1.136  RES 4.576 ± 1.573 |
| 2C: AP-100 pA  CON (n = 16)  SUS (n = 19)  RES (n = 16)  AP-120 pA  CON (n = 16)  SUS (n = 19)  RES (n = 16)  AP-140 pA  CON (n = 16)  SUS (n = 19)  RES (n = 16) | One-way ANOVA  *F* (2, 48) = 8.127, *p* < 0.001  One-way ANOVA  *F* (2, 48) = 7.588, *p* = 0.001  One-way ANOVA  *F* (2, 48) = 8.172, *p* < 0.001 | Bonferroni's  post hoc test  CON vs SUS, *p* = 0.001  SUS vs RES, *p* = 0.020  Bonferroni's  post hoc test  CON vs SUS, *p* = 0.002  SUS vs RES, *p* = 0.017  Bonferroni's  post hoc test  CON vs SUS, *p* = 0.003  SUS vs RES, *p* = 0.005 | CON 5.125 ± 1.052  SUS 11.050 ± 1.220  RES 6.688 ± 0.947  CON 7.313 ± 1.102  SUS 13.210 ± 1.299  RES 8.500 ± 0.975  CON 9.500 ± 1.214  SUS 15.470 ± 1.371  RES 9.813 ± 0.891 |
| 2E: Fluorescence expression of c-Fos^+^ CTB^+^ in PVT  CON (n = 15 slices from 5 mice)  SUS (n = 15 slices from 5 mice) | Unpaired *t* test  *t* = 9.972, *p* < 0.001 |  | CON 40.530 ± 2.531  SUS 100.900 ± 5.650 |
| 2G: c-Fos^+^ CaMKIIα^+^  CON (n = 9 slices from 3 mice)  SUS (n = 9 slices from 3 mice) | Unpaired *t* test  *t* = 4.296, *p* < 0.001 |  | CON 13.560 ± 1.281  SUS 37.000 ± 5.305 |
| 3D: AP-60 pA  Baseline (n = 10)  CNO (n = 10) Washout (n = 10)  AP-80 pA  Baseline (n = 10)  CNO (n = 10) Washout (n = 10)  AP-100 pA  Baseline (n = 10)  CNO (n = 10) Washout (n = 10)  AP-120 pA  Baseline (n = 10)  CNO (n = 10) Washout (n = 10)  AP-140 pA  Baseline (n = 10)  CNO (n = 10) Washout (n = 10) | One-way ANOVA  *F* (2, 27) = 8.677, *p* = 0.001  One-way ANOVA  *F* (2, 27) = 10.570, *p* < 0.001  One-way ANOVA  *F* (2, 27) = 12.990, *p* < 0.001  One-way ANOVA  *F* (2, 27) = 13.220, *p* < 0.001  One-way ANOVA  *F* (2, 27) = 17.740, *p* < 0.001 | Bonferroni's  post hoc test  Baseline vs CNO, *p* = 0.007  CNO vs Washout, *p* = 0.002  Bonferroni's  post hoc test  Baseline vs CNO, *p* = 0.004  CNO vs Washout, *p* < 0.001  Bonferroni's post hoc test  Baseline vs CNO, *p* = 0.002  CNO vs Washout, *p* < 0.001  Bonferroni's post hoc test  Baseline vs CNO, *p* = 0.001 CNO vs Washout, *p* < 0.001  Bonferroni's post hoc test  Baseline vs CNO, *p* < 0.001  CNO vs Washout, *p* < 0.001 | Baseline 5.800 ± 1.083  CNO 10.600 ± 1.921  Washout 5.100 ± 1.038  Baseline 7.000 ± 1.1555  CNO 12.300 ± 0.831  Washout 5.900 ± 1.140  Baseline 8.200 ± 1.123  CNO 13.900 ± 0.706  Washout 7.000 ± 1.174  Baseline 9.400 ± 1.108  CNO 14.900 ± 0.674  Washout 8.300 ± 1.075  Baseline 10.100 ± 0.924  CNO 15.700 ± 0.883  Washout 8.800 ± 0.800 |
| 3E: SIT  VEH (n = 12)  CNO (n = 12) | Unpaired *t* test  *t* = 3.683, *p* = 0.001 |  | VEH 1.625 ± 0.324  CNO 0.389 ± 0.088 |
| 3F: TST  VEH (n = 12)  CNO (n = 14) | Unpaired *t* test  *t* = 2.210, *p* = 0.037 |  | VEH 84.430 ± 11.610  CNO 129.200 ± 15.880 |
| 3G: FST  VEH (n = 11)  CNO (n = 14) | Unpaired *t* test  *t* = 3.934, *p* = 0.001 |  | VEH 61.040 ± 11.470  CNO 133.000 ± 13.450 |
| 3J: Expression of MC4R in NAcsh  AAV-GFP (n = 5)  AAV-shMC4R (n = 5) | Unpaired *t* test  *t* = 2.443, *p* = 0.040 |  | CON 1.000 ± 0.107  CSDS 0.600 ± 0.125 |
| 3K: SIT  VEH + AAV-GFP (n = 7)  VEH + AAV-shMC4R (n = 8)  CNO + AAV-GFP (n = 8)  CNO + AAV-shMC4R (n =8) | Two-way ANOVA  CNO × AAV-shMC4R: *F* (1, 27) = 18.810, *p* < 0.001  AAV-shMC4R: *F* (1, 27) = 9.560, *p* = 0.005  CNO: *F* (1, 27) = 7.549, *p* = 0.011 | Bonferroni's post hoc test VEH + AAV-GFP vs CNO + AAV-GFP, *p* < 0.001  CNO + AAV-GFP vs CNO + AAV-shMC4R, *p* < 0.001 | VEH + AAV-GFP 1.483 ± 0.106  VEH + AAV-shMC4R 1.419 ± 0.084  CNO + AAV-GFP 0.424 ± 0.082  CNO + AAV-shMC4R 1.434 ± 0.170 |
| 3L: TST  VEH + AAV-GFP (n = 7)  VEH + AAV-shMC4R (n = 8)  CNO + AAV-GFP (n = 8)  CNO + AAV-shMC4R (n =8) | Two-way ANOVA  CNO × AAV-shMC4R: *F* (1, 27) = 5.157, *p* = 0.031  AAV-shMC4R: *F* (1, 27) = 2.557, *p* = 0.122  CNO: *F* (1, 27) = 1.670, *p* = 0.207 | Bonferroni's post hoc test VEH + AAV-GFP vs CNO + AAV-GFP, *p* = 0.024  CNO + AAV-GFP vs CNO + AAV-shMC4R, *p* = 0.032 | VEH + AAV-GFP 90.060 ± 17.830  VEH + AAV-shMC4R 99.770 ± 13.370  CNO + AAV-GFP 137.300 ± 5.101  CNO + AAV-shMC4R 93.800 ± 12.290 |
| 3M: FST  VEH + AAV-GFP (n = 7)  VEH + AAV-shMC4R (n = 8)  CNO + AAV-GFP (n = 8)  CNO + AAV-shMC4R (n =8) | Two-way ANOVA  CNO × AAV-shMC4R: *F* (1, 26) = 3.636, *p* = 0.007  AAV-shMC4R: *F* (1, 26) = 6.451, *p* = 0.017  CNO: *F* (1, 26) = 13.810, *p* = 0.001 | Bonferroni's post hoc test VEH + AAV-GFP vs CNO + AAV-GFP, *p* = 0.008  CNO + AAV-GFP vs CNO + AAV-shMC4R, *p* < 0.001 | VEH + AAV-GFP 47.060 ± 7.446  VEH + AAV-shMC4R 19.510 ± 4.535  CNO + AAV-GFP 114.700 ± 23.970  CNO +AAV-shMC4R 29.150 ± 13.080 |
| 4D: AP-40 pA  Baseline (n = 11)  CNO (n = 11) Washout (n = 11)  AP-60 pA  Baseline (n = 11)  CNO (n = 11) Washout (n = 11)  AP-80 pA  Baseline (n = 11)  CNO (n = 11) Washout (n = 11)  AP-100 pA  Baseline (n = 11)  CNO (n = 11) Washout (n = 11)  AP-120 pA  Baseline (n = 11)  CNO (n = 11) Washout (n = 11)  AP-140 pA  Baseline (n = 11)  CNO (n =11) Washout (n = 11) | One-way ANOVA  *F* (2, 30) = 9.889, *p* < 0.001  One-way ANOVA  *F* (2, 30) = 11.680, *p* < 0.001  One-way ANOVA  *F* (2, 30) = 9.477, *p* < 0.001  One-way ANOVA  *F* (2, 30) = 8.856, *p* < 0.001  One-way ANOVA  *F* (2, 30) = 10.390, *p* < 0.001  One-way ANOVA  *F* (2, 30) = 13.340, *p* < 0.001 | Bonferroni's post hoc test  Baseline vs CNO,  *p* < 0.001  CNO vs Washout,  *p* = 0.030  Bonferroni's  post hoc test  Baseline vs CNO,  *p* = 0.0003  CNO vs Washout,  *p* = 0.002  Bonferroni's  post hoc test  Baseline vs CNO, *p* = 0.001  CNO vs Washout, *p* = 0.006  Bonferroni's post hoc test  Baseline vs CNO, *p* = 0.002  CNO vs Washout, *p* = 0.004  Bonferroni's post hoc test  Baseline vs CNO, *p* < 0.001  CNO vs Washout, *p* = 0.003  Bonferroni's post hoc test  Baseline vs CNO, *p* < 0.001  CNO vs Washout, *p* = 0.001 | Baseline 7.818 ± 0.773  CNO 2.727 ± 0.752  Washout 5.909 ± 0.919  Baseline 10.730 ± 0.787  CNO 4.364 ± 1.146  Washout 9.636 ± 1.020  Baseline 13.180 ± 1.069  CNO 6.091 ± 1.351  Washout 12.000 ± 1.265  Baseline 14.450 ± 1.296  CNO 7.000 ± 1.477  Washout 14.000 ± 1.433  Baseline 16.090 ± 1.398  CNO 8.000 ± 1.388  Washout 15.090 ± 1.317  Baseline 17.550 ± 1.231  CNO 8.818 ± 1.354  Washout 7.182 ± 1.236 |
| 4E: SIT  CON + VEH (n = 14)  CON + CNO (n = 14)  SUS + VEH (n = 14)  SUS + CNO (n = 14) | Two-way ANOVA  SUS × CNO: *F* (1, 52) = 8.743, *p* = 0.005  SUS: *F* (1, 52) = 5.694, *p* = 0.021  CNO: *F* (1, 52) = 6.440, *p* = 0.014 | Bonferroni's post hoc test CON + VEH vs SUS + VEH, *p* = 0.001  SUS + VEH vs  SUS + CNO, *p* = 0.001 | CON + VEH  1.495 ± 0.219  CON + CNO 1.397 ± 0.173  SUS + VEH 0.549 ± 0.087  SUS + CNO 1.469 ± 0.182 |
| 4F: TST  CON + VEH (n = 10)  CON + CNO (n = 10)  SUS + VEH (n = 10)  SUS + CNO (n = 10) | Two-way ANOVA  SUS × CNO: *F* (1, 36) = 15.670, *p* = 0.001  SUS: *F* (1, 36) = 7.709, *p* = 0.009  CNO: *F* (1, 36) = 6.201, *p* = 0.018 | Bonferroni's post hoc test CON + VEH vs SUS + VEH, *p* < 0.001  SUS + VEH vs  SUS + CNO, *p* < 0.001 | CON + VEH 79.840 ± 7.230  CON + CNO 92.930 ± 10.540  SUS + VEH 151.200 ± 12.820  SUS + CNO 76.670 ± 12.760 |
| 4G: FST  CON + VEH (n = 10)  CON + CNO (n = 10)  SUS + VEH (n = 10)  SUS + CNO (n = 10) | Two-way ANOVA  SUS × CNO: *F* (1, 36) = 9.308, *p* = 0.004  SUS: *F* (1, 36) = 7.732, *p* = 0.009  CNO: *F* (1, 36) = 10.300, *p* = 0.003 | Bonferroni's post hoc test CON + VEH vs SUS + VEH, < 0.001  SUS + VEH vs SUS + CNO, *p* < 0.001 | CON + VEH 76.990 ± 14.420 CON + CNO 67.110 ± 16.370 SUS+ VEH 158.500 ± 16.650  SUS + CNO 69.520 ± 13.310 |
| 5B: mEPSCs frequency  CON + VEH + VEH  (n = 12)  CON + CNO + VEH  (n =10)  CON + CNO + α-MSH (n = 10)  SUS + VEH +VEH (n = 11)  SUS + CNO + VEH (n = 12)  SUS + CNO + α-MSH (n = 11) | Two-way ANOVA  CNO × α-MSH:  *F* (3, 82) = 5.642, *p* = 0.001  CNO: *F* (1, 82) = 14.600, *p* < 0.001  α-MSH: *F* (3, 82) = 2.954, *p* = 0.037 | Bonferroni's post hoc test  CON + VEH + VEH vs SUS + VEH + VEH, *p* = 0.015  SUS + VEH + VEH vs SUS + CNO + VEH, *p* = 0.017  SUS + CNO + VEH vs SUS + CNO + α-MSH, *p* < 0.001 | CON + VEH + VEH 1.057 ± 0.093  CON + CNO + VEH 1.894 ± 0.256  CON + CNO + α-MSH 1.446 ± 0.130  SUS + VEH + VEH 2.323 ± 0.317  SUS + CNO + VEH 1.053 ± 0.086  SUS + CNO + α-MSH 2.396 ± 0.297 |
| 5C: mEPSCs amplitude  CON + VEH + VEH  (n = 12)  CON + CNO + VEH  (n = 10)  CON + CNO + α-MSH (n = 10)  SUS + VEH + VEH (n = 11)  SUS + CNO + VEH (n = 12)  SUS + CNO + α-MSH (n = 10) | Two-way ANOVA  CNO × α-MSH:  *F* (3, 82) = 5.553, *p* = 0.002  CNO: *F* (1, 82) = 2.341, *p* = 0.130  α-MSH: *F* (3, 82) = 0.095, *p* = 0.963 | Bonferroni's post hoc test  CON + VEH + VEH vs SUS + VEH + VEH, *p* = 0.022  SUS + VEH + VEH vs SUS + CNO +VEH, *p* = 0.038  SUS + CNO + VEH vs  SUS + CNO + α-MSH, *p* = 0.255 | CON + VEH + VEH 8.685 ± 0.822  CON + CNO + VEH 16.290 ± 2.600  CON + CNO + α-MSH 15.300 ± 2.194  SUS + VEH + VEH 16.000 ± 3.205  SUS + CNO + VEH 9.146 ± 0.379  SUS + CNO + α-MSH 12.540 ± 0.940 |
| S2B: Fluorescence expression of c-Fos^+^ in PVT  CON (n = 9 slices from 3 mice)  SUS (n = 9 slices from 3 mice) | Unpaired t test  *t* = 3.751, *p* = 0.002 |  | CON 30.560 ± 4.407  SUS 53.560 ± 4.263 |
| S3B: Fluorescence co-expression of c-Fos^+^ and GAD67 in PVT  CON (n = 9 slices from 3 mice)  SUS (n =9 slices from 3 mice) | Unpaired t test  *t* = 0.203, *p* = 0.842 |  | CON 14.440 ± 0.973  SUS 14.780 ± 1.321 |
| S4B: Distance  VEH (n = 6)  CNO (n = 8) | Unpaired *t* test  *t* = 1.218, *p* = 0.247 |  | VEH 11.820 ± 1.507  CNO 8.788 ± 1.824 |
| S4C: Speed  VEH (n = 6)  CNO (n = 8) | Unpaired *t* test  *t* = 1.234, *p* = 0.241 |  | VEH 0.039 ± 0.005  CNO 0.029 ± 0.006 |
| S4E: Distance  CON + VEH (n = 5)  CON + CNO (n = 5)  SUS + VEH (n = 5)  SUS+ CNO (n = 5) | One-way ANOVA  CNO: *F* (3, 16) = 0.312, *p* = 0.817 | Bonferroni's post hoc test CON +VEH vs SUS +VEH, *p* > 0.999  SUS + VEH vs SUS+ CNO, *p* > 0.999 | CON + VEH 12.700 ± 1.020  CON + CNO 10.750 ± 1.043  SUS + VEH 11.040 ± 1.942  SUS + CNO 11.860 ± 1.998 |
| S4F: Speed CON + VEH (n = 5)  CON + CNO (n = 5)  SUS + VEH (n = 5)  SUS + CNO (n = 5) | One-way ANOVA  CNO: *F* (3, 16) = 0.317, *p* = 0.813 | Bonferroni's post hoc test CON + VEH vs SUS +VEH, *p* > 0.999  SUS + VEH vs SUS + CNO, *p* > 0.999 | CON + VEH  0.042 ± 0.003  CON + CNO 0.036 ± 0.004  SUS + VEH 0.037 ± 0.007  SUS + CNO 0.040 ± 0.007 |
| S5B: SIT  VEH + VEH (n = 8)  VEH + SHU (n = 9)  CNO + VEH (n = 8)  CNO + SHU (n = 10) | Two-way ANOVA  CNO × SHU: *F* (1, 31) = 8.030, *p* = 0.008  SHU: *F* (1, 31) = 8.746, *p* = 0.006  CNO: *F* (1, 31) = 5.508, *p* = 0.026 | Bonferroni's post hoc test VEH + VEH vs CNO + VEH, *p* = 0.001  CNO + VEH vs CNO + SHU, *p* = 0.002 | VEH + VEH 1.642 ± 0.134  VEH + SHU 1.554 ± 0.213  CNO + VEH 0.599 ± 0.098  CNO + SHU 1.532 ± 0.209 |
| S5C: TST  VEH + VEH (n = 10)  VEH + SHU (n = 10)  CNO + VEH (n = 10)  CNO + SHU (n = 10) | Two-way ANOVA  CNO × SHU: *F* (1, 36) = 10.230, *p* = 0.003  SHU: *F* (1, 36) = 11.250, *p* = 0.002  CNO: *F* (1, 36) = 3.710, *p* = 0.062 | Bonferroni's post hoc test VEH + VEH vs CNO + VEH, *p* < 0.001  CNO + VEH vs CNO + SHU, *p* = 0.002 | VEH + VEH 76.810 ± 14.580  VEH + SHU 90.900 ± 9.573  CNO + VEH 161.100 ± 8.817  CNO + SHU 94.280 ± 17.150 |
| S5D: FST  VEH + VEH (n = 10)  VEH + SHU (n = 10)  CNO + VEH (n = 10)  CNO + SHU (n = 10) | Two-way ANOVA  CNO × SHU: *F* (1, 36) = 5.655, *p* = 0.023  SHU: *F* (1, 36) = 13.330, *p* = 0.001  CNO: *F* (1, 36) = 7.599, *p* = 0.009 | Bonferroni's Post hoc test VEH + VEH vs CNO + VEH, *p* = 0.001  CNO + VEH vs CNO + SHU, *p* = 0.002 | VEH + VEH 71.400 ± 14.150  VEH + SHU 65.870 ± 17.410  CNO + VEH 159.400 ± 7.617  CNO + SHU 84.460 ± 17.040 |
| S6B: D1-SIT  CON + VEH (n = 7)  CON + CNO (n = 7)  SUS + VEH (n = 7)  SUS +CNO (n = 7) | Two-way ANOVA  CSDS × CNO: *F* (1, 22) = 0.571, *p* = 0.458  SUS: *F* (1, 22) = 0.389, *p* = 0.539  CNO: *F* (1, 22) = 45.480, *p* < 0.001 | Bonferroni's post hoc test CON + VEH vs SUS + VEH, *p* < 0.001  SUS + VEH vs SUS + CNO, *p* > 0.999 | CON + VEH 1.257 ± 0.108  CON + CNO 1.472 ± 0.210  SUS + VEH 0.325 ± 0.156  SUS + CNO 0.304 ± 0.134 |
| S6C: D3-SIT  CON + VEH (n = 7)  CON + CNO (n = 7)  SUS + VEH (n = 7)  SUS +CNO (n = 7) | Two-way ANOVA  SUS × CNO: *F* (1, 22) = 2.041, *p* = 0.167  SUS: *F* (1, 22) = 1.225, *p* = 0.280  CNO: *F* (1, 22) = 31.770, *p* < 0.001 | Bonferroni's post hoc test CON + VEH vs SUS + VEH, *p* < 0.001  SUS + VEH vs SUS + CNO, *p* = 0.151 | CON + VEH 1.191 ± 0.151  CON + CNO 1.145 ± 0.192  SUS + VEH 0.188 ± 0.069  SUS + CNO 0.548 ± 0.145 |

**Original scan of key western blots**


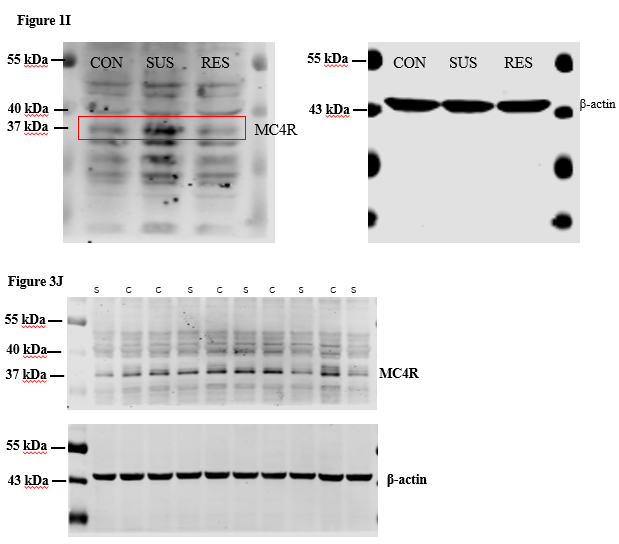


**Original blots for Figure 1I, 3J.**
