# Supplementary figures and images for: A thalamic circuit facilitates stress susceptibility via melanocortin 4 receptor‐mediated activation of nucleus accumbens shell
Source: CNS Neurosci Ther. 2022 Dec 12;29(2):646–58. doi: 10.1111/cns.14046 (PMC9873525; doi:10.1111/cns.14046)

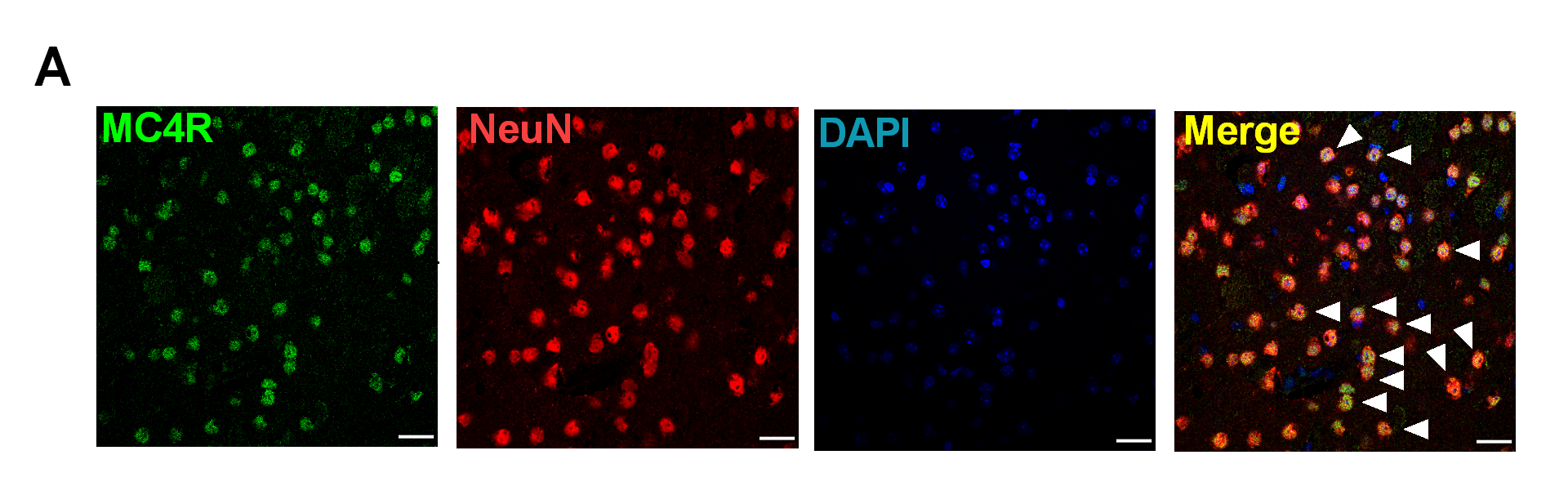

Supplement: Supplementary file 2 — Figure S1. [file CNS-29-646-s002.tif]

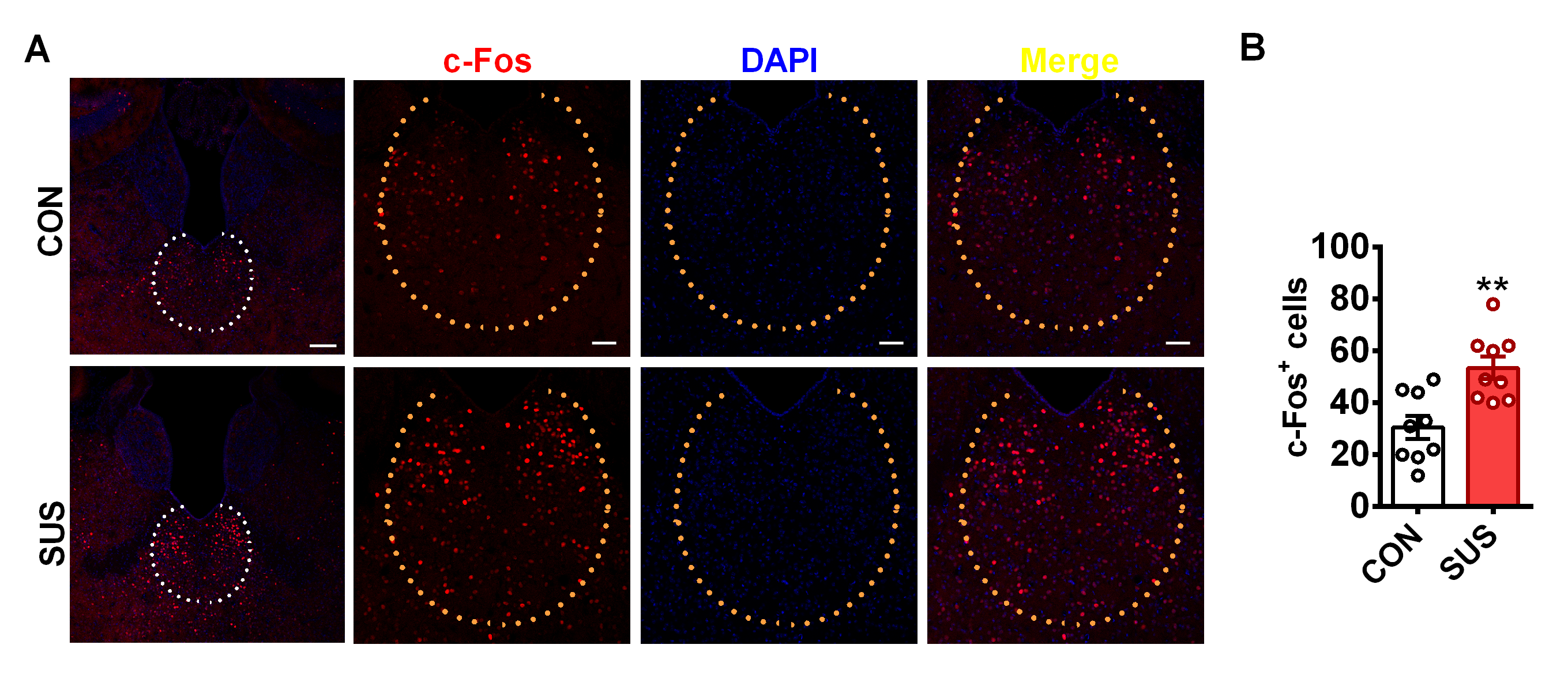

Supplement: Supplementary file 3 — Figure S2. [file CNS-29-646-s003.tif]

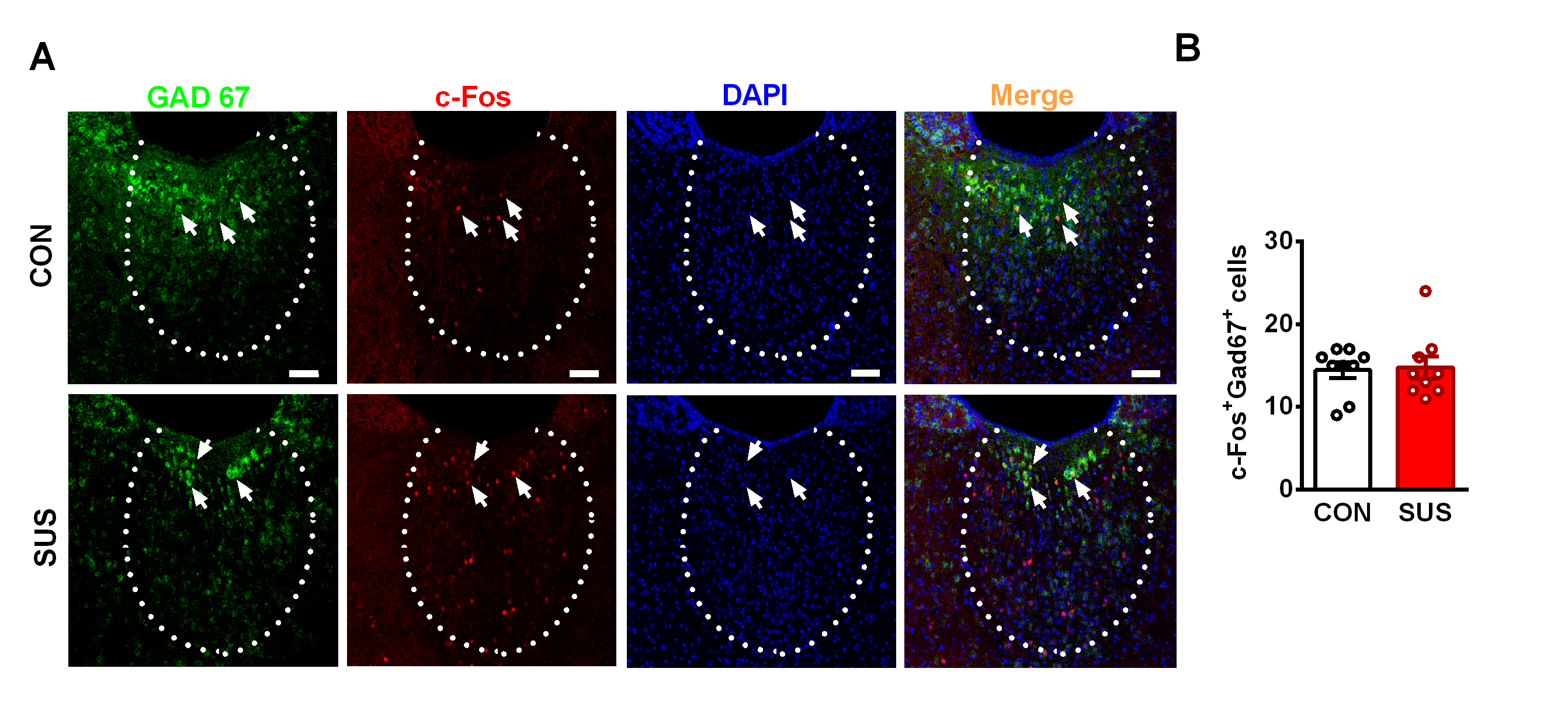

Supplement: Supplementary file 4 — Figure S3. [file CNS-29-646-s005.tif]

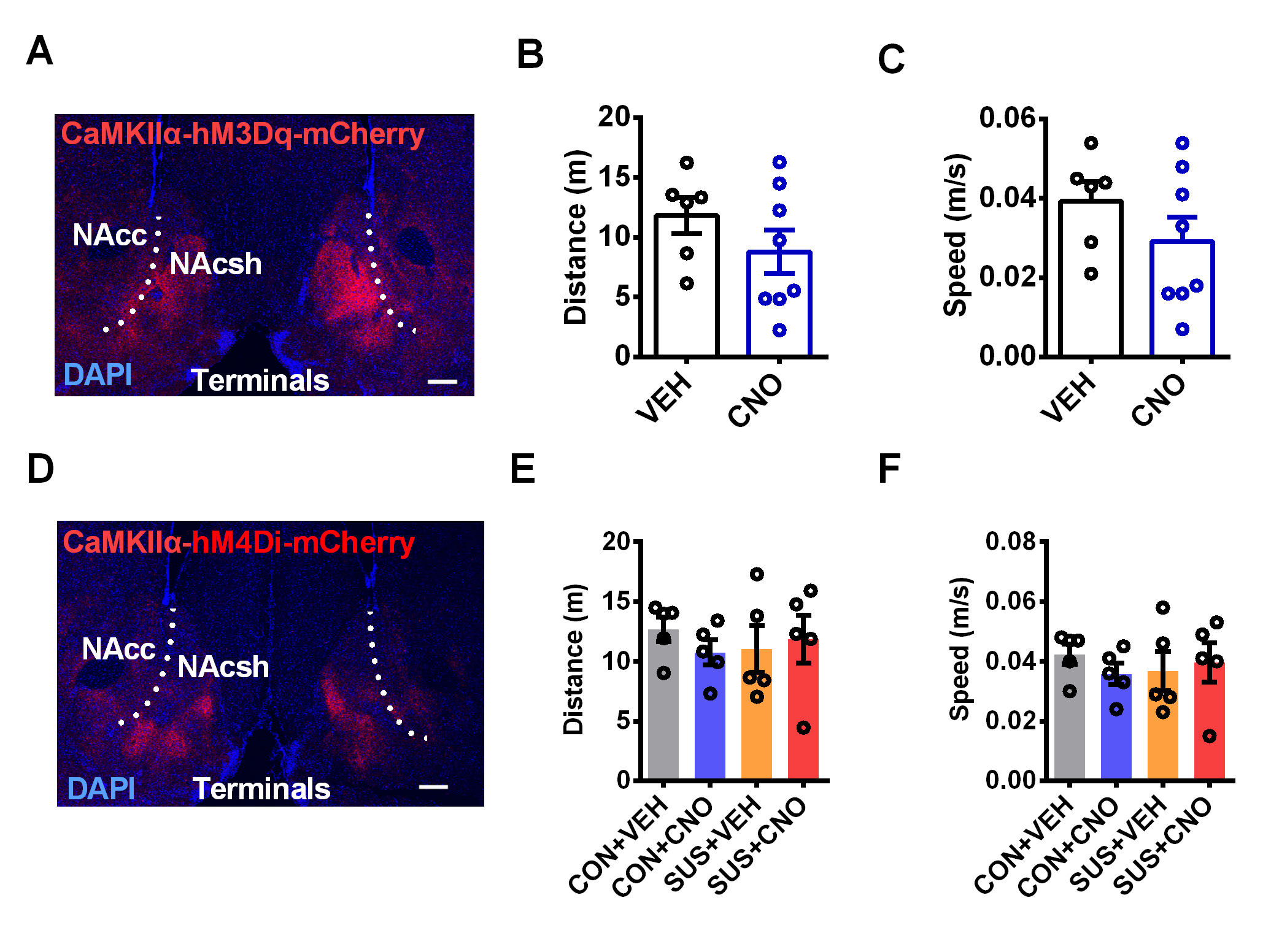

Supplement: Supplementary file 5 — Figure S4. [file CNS-29-646-s004.tif]

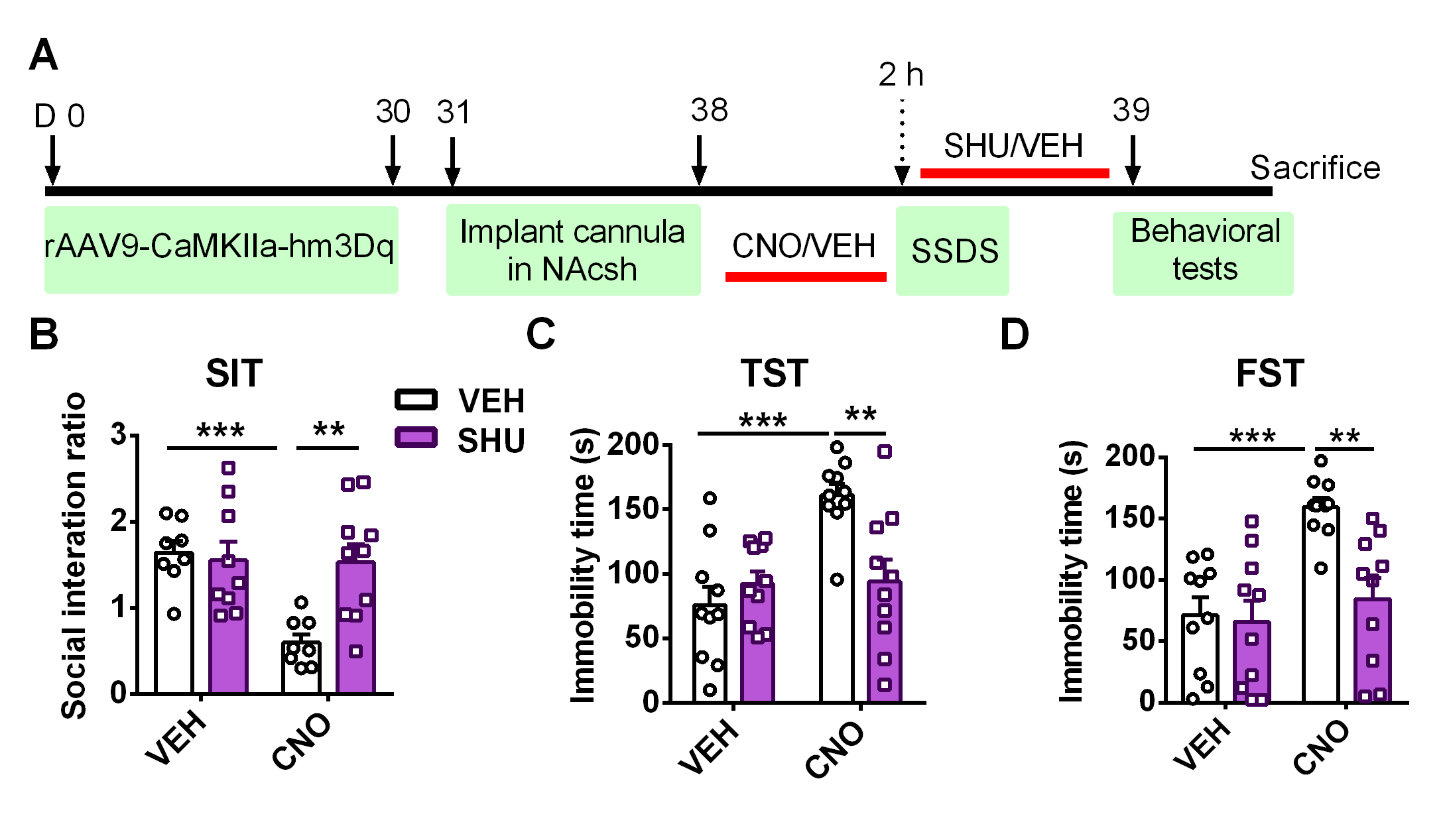

Supplement: Supplementary file 6 — Figure S5. [file CNS-29-646-s001.tif]

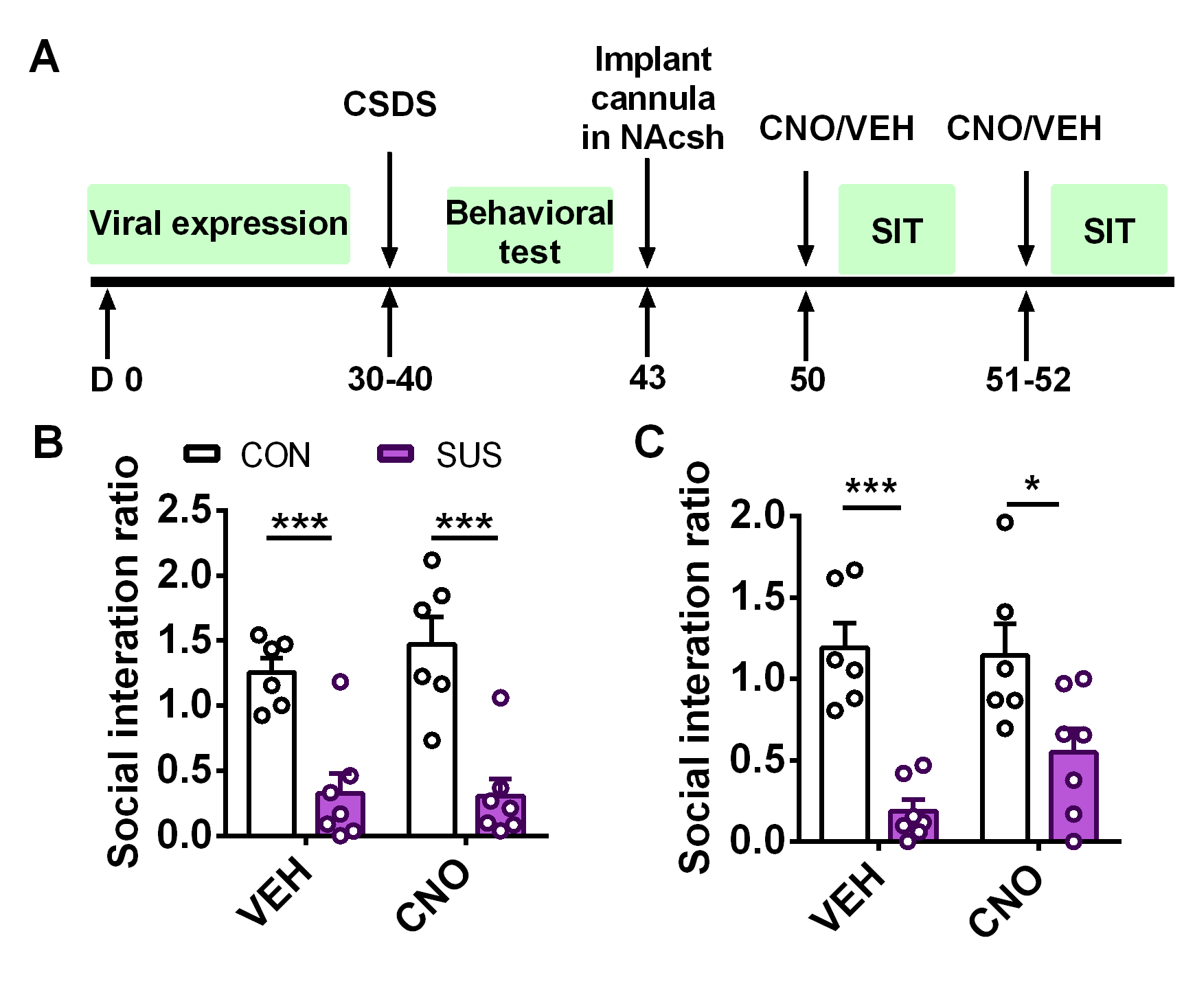

Supplement: Supplementary file 7 — Figure S6. [file CNS-29-646-s007.tif]
